# Telling the whole story in a 10,000-genome world

**DOI:** 10.1186/1745-6150-6-34

**Published:** 2011-06-30

**Authors:** Robert G Beiko

**Affiliations:** 1 Faculty of Computer Science, Dalhousie University, 6050 University Avenue, Halifax, NS B3H 1W5 Canada

## Abstract

**Background:**

Genome sequencing has revolutionized our view of the relationships among genomes, particularly in revealing the confounding effects of lateral genetic transfer (LGT). Phylogenomic techniques have been used to construct purported trees of microbial life. Although such trees are easily interpreted and allow the use of a subset of genomes as "proxies" for the full set, LGT and other phenomena impact the positioning of different groups in genome trees, confounding and potentially invalidating attempts to construct a phylogeny-based taxonomy of microorganisms. Network and graph approaches can reveal complex sets of relationships, but applying these techniques to large data sets is a significant challenge. Notwithstanding the question of what exactly it might represent, generating and interpreting a Tree or Network of All Genomes will only be feasible if current algorithms can be improved upon.

**Results:**

Complex relationships among even the most-similar genomes demonstrate that proxy-based approaches to simplifying large sets of genomes are not alone sufficient to solve the analysis problem. A phylogenomic analysis of 1173 sequenced bacterial and archaeal genomes generated phylogenetic trees for 159,905 distinct homologous gene sets. The relationships inferred from this set can be heavily dependent on the inclusion of other taxa: for example, phyla such as Spirochaetes, Proteobacteria and Firmicutes are recovered as cohesive groups or split depending on the presence of other specific lineages. Furthermore, named groups such as *Acidithiobacillus, Coprothermobacter *and *Brachyspira *show a multitude of affiliations that are more consistent with their ecology than with small subunit ribosomal DNA-based taxonomy. Network and graph representations can illustrate the multitude of conflicting affinities, but all methods impose constraints on the input data and create challenges of construction and interpretation.

**Conclusions:**

These complex relationships highlight the need for an inclusive approach to genomic data, and current methods with minor alterations will likely scale to allow the analysis of data sets with 10,000 or more genomes. The main challenges lie in the visualization and interpretation of genomic relationships, and the redefinition of microbial taxonomy when subsets of genomic data are so evidently in conflict with one another, and with the "canonical" molecular taxonomy.

**Reviewers:**

The manuscript was reviewed by William Martin, W. Ford Doolittle, Joel Velasco and Eugene Koonin.

## Background

Our current understanding of microbial diversity, function, and ecology owes a great deal to the ready availability of genome sequence data for a multitude of microorganisms. Genomes, predicted genes and functional annotations can be easily acquired from many online databases, and many of these offer multiple methods of automated retrieval. With a small amount of coding skill, a researcher can couple the acquisition of data with an analytical pipeline to produce up-to-date information about the evolution and function of an ever-expanding list of organisms. The sampling of diversity is neither random nor representative, and the current list of available genomes reflects particular interests in medically or industrially important pathogens, extremophiles, and organisms with important economic value (e.g., agricultural pests and commensals). Nonetheless, the breadth of sampling is impressive, with members of 36 bacterial and archaeal phyla sequenced by the end of 2009, and the unequal representation of different lineages allows comparative studies to be carried out at shallow and deep levels. Furthermore, sequencing projects are now targeting "gaps" in genomic diversity, directly via the GEBA project [1] and indirectly through various environmental genomic studies [2,3].

Major phylogenomic studies have used large genomic data sets to construct phylogenies or other similarity-based relationships among genomes, with the goal of inferring patterns and/or processes with as comprehensive a data set as possible. Some analyses have claimed that their recovered structures (tree or network) are an explicit representation of organismal relationships (e.g., a Tree of Life), while others have presented similar structures only as a representation of genetic affinities with no attached claim of having found the "true" relationships among species. The most familiar approaches involved the use of a subset (typically of size 1-50) of genes that are widespread or universal in their distribution, with phylogenetic analysis based on a concatenated alignment of these genes. For example, the hypothesis that all known organisms can be split into three domains (Eukarya, Archaea and Bacteria) was originally based on the analysis of small-subunit ribosomal DNA (SSU) sequences [4,5]. Concatenated alignment or "supermatrix" approaches have been used to propose deep phylogenetic relationships for all three domains of life [6-9]. Another class of approaches used to infer genomic relationships are based on the abstraction of genes as a set of binary presence/absence criteria. Phylogenetic profiles describe each genome in terms of the presence and absence of proteins from different homologous or orthologous gene sets: early genome trees based on this type of representation were largely congruent with reference trees based on marker genes, which was taken as evidence that genomic data could be used to infer higher-order relationships among groups of organisms [10,11]. A modified version of this approach served as the basis of the Ring of Life hypothesis [12], and variations that use quantitative rather than qualitative presence/absence information have revealed interesting alternative genetic affinities, especially when reweighting schemes are used [13]. Lienau et al. [14] combined evidence from protein phylogenetic data and gene presence/absence information into a "mega-matrix", analogous to a large multiple sequence alignment, to construct a parsimony tree which was claimed to be similar to that of [8]. Yet another type of analysis considers many individual gene trees, either combining them into a single "supertree" that reflects frequently observed phylogenetic patterns [15-17], or using statistics to summarize and assess the support of different topological features [18,19].

Many algorithms can be used to execute the necessary steps of a phylogenomic analysis. The customary first step after the retrieval of annotated genome sequences is the inference of homologous relationships using an algorithm such as BLAST [20]. Naïve pairwise comparison of sequences using BLAST immediately imposes a quadratic scaling of the analysis with increasing amounts of data: a full all-versus-all comparison of 1000 sequences using BLAST requires 1,000,000 pairwise comparisons, but the same analysis on 10,000 sequences requires 100,000,000 comparisons. Programs such as CD-HIT [21] and UCLUST [22] aim to avoid all-versus-all sequence comparisons by building clusters of similar sequences on the fly, and using only a single "seed" sequence from each cluster for comparative purposes. In this way, seed sequences are intended to serve as proxies for non-seed sequences in their clusters, with a concomitant trade-off of accuracy to realize increased performance. Other strategies such as FastBLAST [23] aim to minimize the number of comparisons performed, although such methods are still time-consuming when data sets are large. Once these initial comparisons have been completed, many options are available for sequence alignment and phylogenetic inference. These methods typically aim to maximize a function that describes the fit of a given alignment or tree instance to the data, using a model of sequence substitution to represent rates of evolutionary change between different types of nucleotide or amino acid residue. Exhaustive alignment and phylogeny approaches cannot be applied to data sets with > 10 sequences, but heuristic approaches have been developed that sacrifice the guarantee of optimality for the benefit of being able to run on much larger sequence data sets. Current methods have been applied to data sets comprising over 100,000 sequences [24]. However, the sacrifices made by some methods may not be acceptable, and other types of analysis such as phylogenetic network reconstruction and inference of lateral genetic transfer have fewer heuristic algorithms available [25].

Scaling up analyses to the currently available 1000 genomes and future sets comprising > 10,000 genomes will obviously depend on the availability of methods that can efficiently analyze large data sets. Such methods will need to exploit recurring patterns in genomic data, with the goal of reducing the total amount of data that needs to be subjected to intensive analysis, for instance by identifying and removing redundant gene sequences or even entire genomes. A final challenge in large-scale phylogenomics is the interpretation of large sets of trees and other data structures: visual summaries are vital to understanding, but inspection of a phylogenetic tree or network with even 100 leaves can be a daunting task. The goal of this paper is to examine the currently available set of sequenced microbial genomes for evidence of repeated structure, perform a rapid phylogenomic analysis of these genomes in a manner similar to previous large-scale studies (e.g., [16,18]) using newly available algorithms, and explore different techniques for visualizing conflicting relationships, either across the entire data set or with a particular focus on a taxonomic group of interest. By analyzing a set of over 1000 genomes using techniques described above and new tools and techniques from the literature, we can anticipate the challenges that will arise with much larger genomic data sets comprising both novel phyla and many representatives from intensively sampled taxonomic groups.

## Results and Discussion

### Sampling of taxonomic groups and "protein space": is there any evidence for saturation?

**Figure 1:**
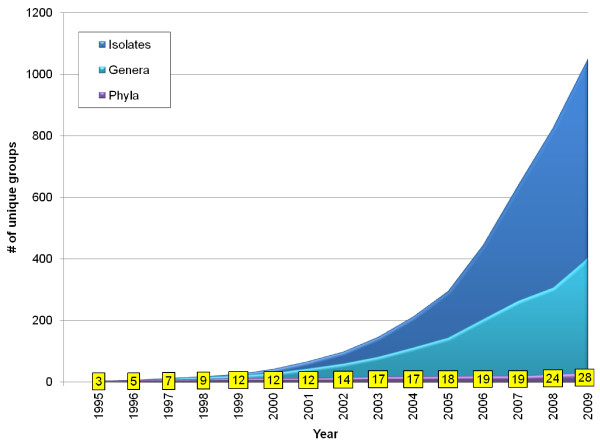
**Accumulation of new genomes and taxonomic groups from 1995-2009**. The total number of sequenced genomes available at NCBI is shown in dark blue, the total number of distinct genera in light blue, and the total number of phyla in purple. Numbers in yellow boxes correspond to counts of phyla from the purple curve.

Although the sampling of microbial genomes is heavily biased in favour of certain types of organisms [26,27], the taxonomic diversity of sequenced genomes continues to increase (Figure 1). While well-represented phyla such as the Proteobacteria and Firmicutes continue to grow, the period from 2007-2009 saw a 50% increase in the number of represented phyla, with the addition of Deferribacteres, Dictyoglomi, Elusimicrobia, Gemmatimonadetes, Korarchaeota, Nitrospirae, Synergistetes and Verrucomicrobia, and a bacterium (*Thermobaculum terrenum*) that is not yet formally assigned to a phylum. Some of these phyla may be quite limited in their genetic diversity; for example, the genus *Dictyoglomus *is the only known representative of its phylum thus far [28]. However, other phyla such as Synergistetes [29,30] are known to harbour considerably more diversity, and the Verrucomicrobia are an extremely diverse group with few cultivated representatives. The Verrucomicrobia also contain proteins such as tubulins that are rare or absent from other bacterial phyla [31], suggesting that many proteins with homology to no currently known protein still remain to be discovered in the mass of as-yet-uncultured and unsequenced microbial diversity. The number of distinct genera has doubled since 2006, as has the total number of sequenced isolates. Even well-sampled groups have seen substantial increases: in 2008-2009, the number of proteobacterial genera with sequenced representatives increased from 183 to 229.

**Figure 2:**
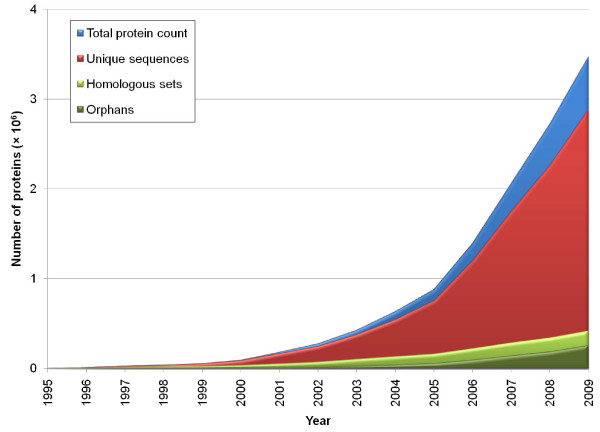
**Accumulation of proteins encoded by sequenced genomes**. The count of proteins (in millions) is shown for different nested criteria of uniqueness: the total count of annotated proteins is shown in blue, the count of unique sequences in red, the total count of homologous sets in light green, and the subset of these that are orphans (no detectable homologs at a BLAST threshold of 1 × 10^-10^) in dark green.

Since LGT can produce a distribution of proteins that is patchy across the SSU rDNA tree, taxonomic groups, and any other hierarchical relationship that might be proposed [32-34], it is not necessarily the case that the first sequenced representative of a genus or phylum will contain many proteins that are "new" in the sense of having homology to no presently known proteins. Relative to an established database of sequences, a newly sequenced gene might be considered novel under several different criteria. The weakest condition of novelty accepts all genes from a newly sequenced genome as novel, regardless of their similarity to existing sequences. At the end of 2009, there were a total of 3,478,477 annotated proteins in bacterial and archaeal genomes. Some newly annotated proteins are 100% identical to an existing protein, in which case only one needs to be included in the sequence alignment and phylogenetic inference steps, while the others can be restored in the appropriate location in the final tree or network. Removing all but one copy from each set of identical sequences reduces the data set by 16.9%, to 2,891,231 unique proteins (Figure 2). Since the number of BLAST comparisons in a naïve all-versus-all analysis increases with the square of the number of sequences, filtering unique sequences would reduce the number of pairwise comparisons by 30%. A homologous set is defined theoretically as a group of one or more proteins in which each protein is homologous with every other protein in the set; such sets are assumed to be maximal, in the sense that all homologous pairs of proteins are assigned to the same homologous set. In practice, empirical, putative homologous sets inferred from sequence data rarely satisfy both of the above criteria, since homology is not always detectable from sequence similarity, and fusion proteins (among others) produce "partial homology" relationships that make perfect sets impossible to define. On this data set, a simple BLAST threshold criterion used to infer homology based on sequence similarity identified 418,214 homologous sets of proteins; over half of these sets (255,417) were orphan proteins in homologous sets of size 1. The increase in protein numbers parallels that of genomes outlined above, with counts of total proteins, unique proteins, homologous sets and orphans more than doubling in the period 2005-2009.

**Figure 3:**
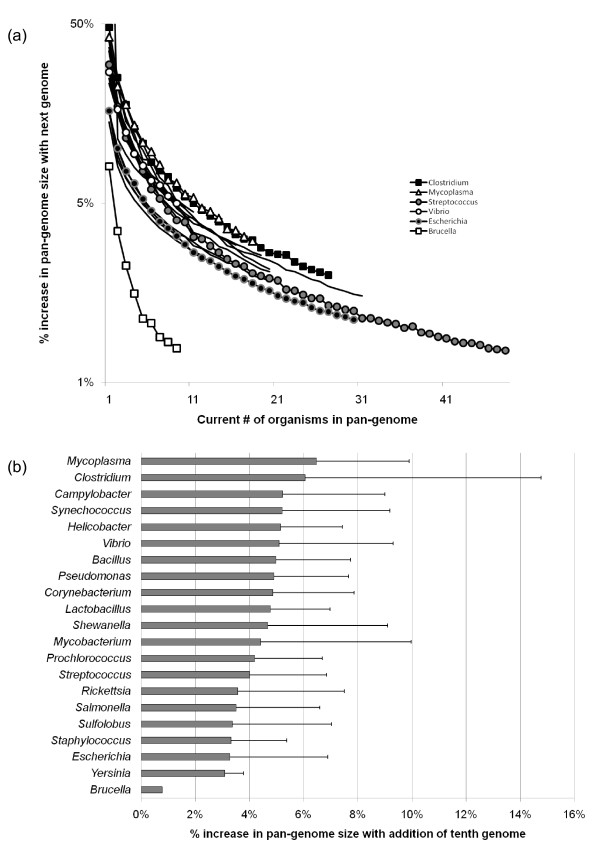
**Relationship between sampling depth of congeners and pangenome size**. (a) Percent increase in pan-genome size (number of proteins assigned to previously unobserved homologous sets vs. total number of proteins) as the number of sequenced members of a genus increases from ***x ***(current # of organisms) to ***x*** + 1. Markers indicate average values for each ***x*** over ten randomized replicate sets for six selected genera, while lines without markers show results for another fifteen genera. (b) Percent increase for 21 genera as the number of sequenced representatives increases from nine to ten. Gray bars indicate average values over ten replicates, while error bars indicate the standard deviation.

Sequencing many isolates from a given species or genus will tend to add less genetic diversity to the existing pool as more examples accumulate, but there is no sign of saturation in the number of "interesting" (distinct and/or novel) proteins here either. The concept of "open" versus "closed" pan-genomes was introduced by [35], and bears directly on the question of the net result of sampling additional genomes within a given taxonomic unit whose members are expected to display some level of genomic similarity that is due to recent shared ancestry and common descent (typically, genus or named species). Figure 3a shows the increase in the number of homologous sets of proteins as additional members of specific genera are sampled, for 20 genera with at least ten sequenced representatives each. Within this group, *Brucella *is a notable outlier (also noted by [36]) with the tenth added genome increasing the count of homologous sets by < 1%. This suggests that the sampled members of *Brucella *are largely clonal, with little variation in gene content. The other 19 genera show similar patterns of homologous set accumulation with increased sampling, although the precise gain for any given number of genomes varies across genera and also depends on which genomes are sampled. For example, adding a tenth genome to an existing set of nine increases the number of homologous sets by over 6% in genus *Mycoplasma *(Figure 3b), but only 3% in *Escherichia*, a genus noted for its variation in gene content [37]. *Mycoplasma *and *Clostridium *stand out as genera with particularly large variation in gene content: this is not necessarily surprising for *Clostridium*, a highly heterogeneous group that is likely in need of further taxonomic refinement [38]. The variation in homologous set gain arising from different random orderings of genome addition is also informative about the spread of genetic variation within a genus. Heavily biased sampling of a few different groups (e.g., named species) can produce large variation, since some added genomes will be nearly identical to others already in the set, while others will represent new subgroups and add many new homologous gene sets. Genera with large standard deviations such as *Clostridium *and *Mycobacterium *have imbalanced internal structures as the sampled genomes are dominated by a few pathogenic species such as *C. botulinum *and *M. tuberculosis*, and genomes in both groups have a wide range of gene counts: different amounts of novelty will result if the tenth added genome is *M. leprae *(1284 genes) or *M. smegmatis *(3490 genes). *Synechococcus *is a particularly perverse case where multiple phylogenetically distinct groups have been assigned to the same genus [39]. Although sequencing additional members of any genus yields diminishing numbers of additional gene families, these examples show that even after a few dozen representatives of a given genus have been sequenced, there is still more genetic novelty in that group that has yet to be observed. Given the amount of novel genetic information in new genomes and the increasing rate at which genomes are being sequenced, there is consequently no reason to suspect that the rate of accumulation of novel genes will decrease in the near future.

### Can certain genes or genomes serve as proxies for the complete set?

Trees based on SSU rDNA, and to a lesser extent other marker genes such as RpoB [40,41] have been used as the basis for evolutionary and taxonomic classification of microbes. This strategy implies the acceptance that a particular gene or set of genes among thousands can serve as a proxy for the evolutionary relatedness of organisms [42]. In the case of SSU rDNA and informational proteins in general, the soundness of this claim has been put forward based on supposed essentiality and recalcitrance to LGT [43,44], although empirical evidence for transfer of even universal ribosomal proteins has been shown [45,46]. In addition to these "genetic proxies", many studies have aimed to use simplify the analyzed data set by using "genomic proxies", in which a small subset of the available members of a given taxonomic group are chosen to represent the entire group.

Proxy-based approaches reduce the size of the data set that needs to be considered, often by one or more orders of magnitude, thereby expanding the range of algorithms that can be applied, and simplifying the visualization and interpretation of results. Such methods, however, necessarily fall short when the fundamental assumptions of proxy approaches are violated. Broad generalizations based on SSU data are only valid to the extent that SSU rDNA faithfully tracks organismal and genomic patterns of inheritance and relatedness. Although many different approaches have produced results that are claimed to be "similar" to the SSU rDNA tree, it is clear that (i) even though the standards for its analysis are higher than for many other genes due to the availability of many examples and the careful mapping of structural information onto reference alignments, the analysis of SSU rDNA is still subject to the usual biases that confound phylogenetic analysis [47]; (ii) the evolutionary histories of many other genes do not match that of SSU rDNA; and (iii) genome-level relationships are more correctly represented with a network or graph, and genome networks will differ considerably from a network based only on SSU sequences, which would likely contain relatively few, localized reticulations that reflect phylogenetic uncertainty. As for the use of genome-level proxies, the highly reticulated nature of relationships among prokaryotes means that choosing one microorganism to stand in for others cannot be done naïvely (for instance, based only on species or genus-level taxonomy), as many important linkages will be missed.

**Figure 4:**
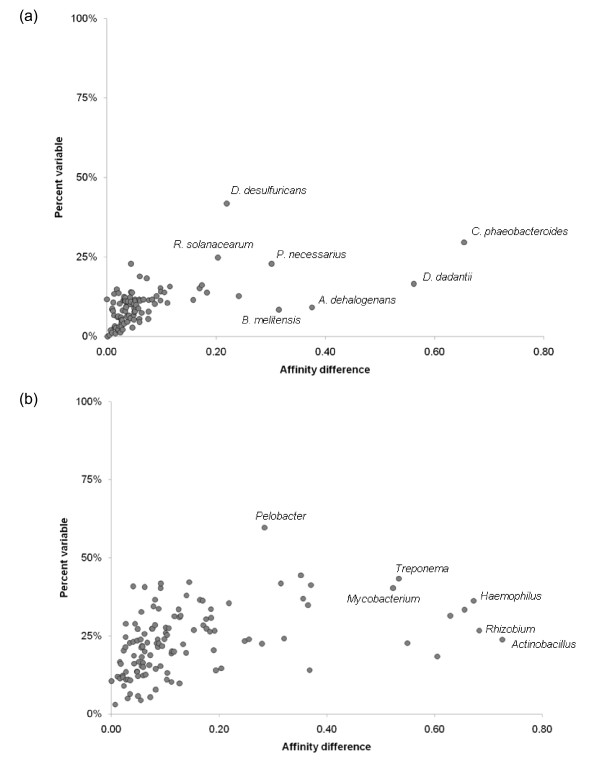
**Affinity differences between randomly selected genome pairs**. The relationship between the percentage of encoded proteins that are not shared between two genomes (% variable) and the difference between taxonomic profiles of best BLAST matches is shown for randomly chosen pairs of conspecific (Figure 4a) and congeneric (Figure 4b) organisms. Highly divergent pairs are identified by their species or genus name.

Members of the same named species and different species within the same genus (Figure 4) can differ dramatically in terms of their common gene inventory, and the taxonomic affinities of the genes they contain. The similarity of sequence-based taxonomic affinities was assessed for many pairs of conspecific and congeneric genomes, both in terms of the affinity of each member of the pair for the other, and in terms of the overall similarity of their patterns of taxonomic matches to each other and to other taxonomic groups. The latter was assessed by building proportional counts of affinities for proteins in each genome to proteins of other taxonomic groups, and computing the Euclidean distance between these sets of counts for the pair of genomes under consideration. The affinity differences shown in Figure 4 reflect the degree to which these patterns of matching differ from one another (see Methods). The majority of genomes from paired conspecific organisms (Figure 4a) have 85% or more homologous gene sets in common, but many pairs nonetheless show considerable differences in their affinities. The two members of *Chlorobium phaeobacteroides *(affinity difference = 0.65) show different affinities to other members of their own family: 53.2% of encoded non-orphan proteins in *C. phaeobacteroides *strain BS1 match best to genus *Prosthecochloris*, whereas proteins in strain DSM 266 tend to best match the congener *C. limnicola *(39.0%) and genus *Pelodictyon *(32.4%), the other member of the "*Chlorobium*/*Pelodictyon *group" within family Chlorobiaceae. While the large proportion of shared genes in the chosen *B. melitensis *pair is expected, the level of affinity difference is high but due largely to differential affinities to other species in genus *Brucella*, including *B. abortus, B. microti, B. ovis, B. suis *and *B. canis*. Similarly, differences in affinities within the delta-proteobacterial species *Anaeromyxobacter dehalogenans *largely reflect different proportions of best matches to *Anaeromyxobacter *species K and Fw109.

At the genus level, there is considerably more variation in gene content and affinity (Figure 4b). The genomes from species *Actinobacillus pleuropneumoniae *and *Actinobacillus succinogenes *have stronger affinities to members of genera *Haemophilus *and *Mannheimia*, respectively, than they do to each other. Affinity differences of a similar nature are seen for *Rhizobium leguminosarum *(strongest affinity to *Agrobacterium*) and *Rhizobium *sp. NGR234 (strongest affinity to *Sinorhizobium*), and for other prominent groups such as *Haemophilus*. Although the affinity differences here are determined largely by differential associations at the next-highest taxonomic rank, the variable genome in each of the above cases consists of genes with affinities to many different taxonomic groups in other families, classes and phyla: these differences are potentially very important but may be missed by proxy-or aggregation-based approaches.

### Data set reduction strategies and genome phylogeny

A common approach to inferring putative evolutionary relationships among genomes is to build trees or networks based on pairwise distances between genomes. Distance-based approaches are popular because they can be very efficient, with the widely used neighbor-joining algorithm having a time complexity of O(n^3^), meaning the computation time scales cubically with increasing numbers of input sequences [48]. The first distance-based genome phylogenies were based on the proportion of homologous sets that are shared in common between different pairs of genomes [10]. Refinements of this basic approach include distances based on the similarity of all putatively orthologous loci between each pair of genomes rather than the binary presence/absence criterion. Conditioned reconstruction [49] is a gene content-based approach that aims to correct for the tendency of small genomes to share many loci in common (a large proportion of which is typically a "universally conserved core": [50,51]), and was used to propose a novel ring-like structure to describe genomic relationships. Conditioned reconstruction approaches are highly dependent on the choice of conditioning genome, although alternatives have been proposed that eliminate the need for a choice of specific genome [52,53]. Other artifacts include the tendency of small genomes to group with one another due to independent loss of similar sets of loci [11], which can be remedied to some extent by normalization for genome size and correction for unequal evolutionary rates [54]; and the tendency of methods to yield "phylogenetic compromises" in the face of conflicting signals that arise due to LGT [55].

**Figure 5:**
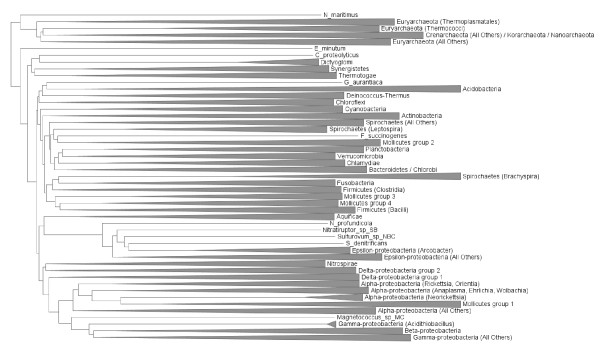
**Normalized BLASTP-based distance tree for 1173 completely sequenced genomes**. Cohesive taxonomic groupings are represented using triangles, while cohesive subgroups separated from other members of their parent group are identified with parentheses in the tip label.

If distances between genomes can be calculated efficiently, then genome phylogeny approaches, even though subject to biases and certain oversimplifying assumptions, can be used to assess the impact of including or excluding particular taxa on the overall inferred relationships. Given the prominence of LGT and statistical artifacts, a genome phylogeny cannot be taken as an authoritative statement about organismal or genomic relationships, nor does it constitute proof that such relationships even have meaning [56]. The implied relationships can nonetheless yield insights into genomic affinities, especially in cases where the positioning of a taxonomic group contradicts other data sources such as SSU rDNA or concatenated informational proteins, or the position of a group changes depending on which other groups are included or excluded, or the weightings of different genes are changed [13]. Figure 5 shows a normalized BLASTP-based genome phylogeny constructed for 1073 genomes, rooted between the Bacteria and Archaea and aggregated into taxonomically cohesive groups up to the level of phylum. Although named taxonomic groups show a great deal of cohesion in this tree, there are some unusual features and intriguing exceptions. For example, the bacterial phyla Nitrospirae and Aquificae and a subset of Mollicutes are found in different positions within the Proteobacteria. Aquificae are sister to the Epsilon-proteobacteria, a relationship that has been proposed elsewhere, while the Mollicutes are sister to a number of alpha-proteobacterial genera that are also reduced in size. The Nitrospirae do not appear as a separate phylum, but rather as a close affiliate of the Delta-proteobacteria, an association reported elsewhere based on 16S analysis [57] and conserved gene order [58]. Another anomaly is the Gamma-proteobacterium *Shewanella denitrificans*, which branches within the Epsilon-proteobacteria as sister to genus *Arcobacter*, when the remainder of its congeners branch as expected within the Gamma-proteobacteria. The Spirochaetes are split into three groups, with genus *Brachyspira *as sister to the Fusobacteria and *Leptospira *as sister to group comprising another set of Mollicutes (*Mycoplasma *and *Ureaplasma*) and cellulolytic *Fibrobacter succinogenes*, the lone representative of phylum Fibrobacteres in the set. Another unusually positioned genome in the set is the Firmicute (class Clostridia) *Coprothermobacter proteolyticus*, which branches with other major thermophile-containing phyla Dictyoglomi, Synergistetes, and Thermotogae near the base of the bacterial tree. It is unclear whether this association is driven by legitimate genetic affinities, or is simply a consequence of a lack of affinity of these groups for other phyla in the tree, and an analog of the well-studied long-branch attraction artifact [59] in phylogenetics.

**Figure 6:**
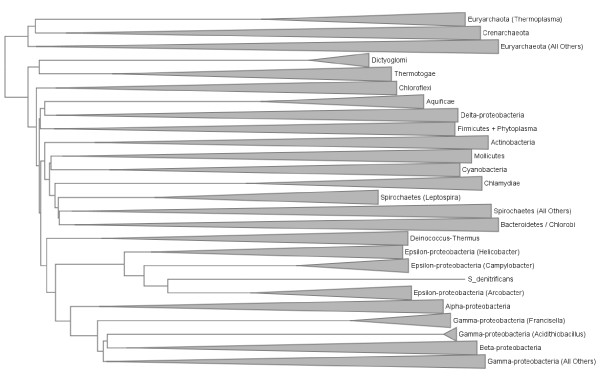
**Normalized BLASTP-based distance tree for 298 completely sequenced genomes from 149 distinct genera**. Groups are summarized as in Figure 5.

To investigate the consequences of using a smaller data set, a tree was constructed from the same starting set of pairwise distances, but including only those genera with two or more sequenced representatives. For each of these genera, the two largest representatives were chosen, with the added condition that both genomes must be members of different named species if possible. The tree for the resulting set of 298 genomes is shown in Figure 6. While many major lineages from Figure 5 are now missing, several differences in the position of the lineages that remain are apparent. A striking difference is the separation of the Delta-proteobacteria from the other members of their phylum; instead they appear as sisters to the Aquificae (represented here by the lone genus *Sulfurihydrogenibium*). Thermotogae and Dictyoglomi remain together at the base of the bacterial tree; the branching order of other phyla is changed, but support for relationships above the phylum level is notoriously weak and unstable [15]. In the absence of Fusobacteria, genus *Brachyspira *rejoins the main lineage of Spirochaetes, although *Leptospira *remains as a separate group. *S. denitrificans *remains as sister to *Arcobacter *in the Epsilon-proteobacteria.

**Figure 7:**
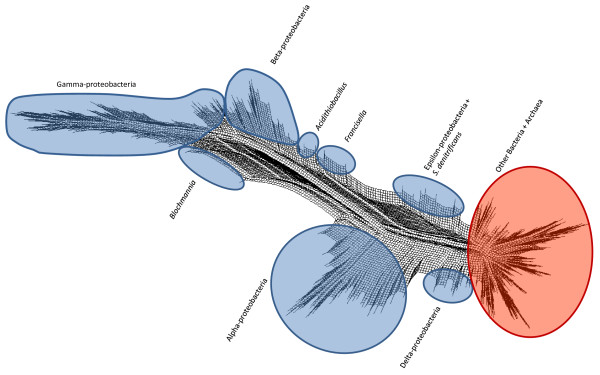
**Neighbor-net showing relationships among 298 completely sequenced genomes (focused on Proteobacteria)**. Proteobacterial divisions and outlying groups are indicated with separate blue regions.

**Figure 8:**
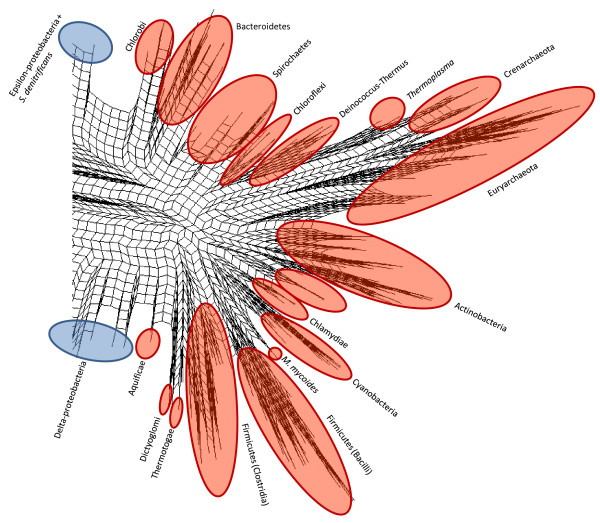
**Neighbor-net showing relationships among 298 completely sequenced genomes (focused on other bacterial phyla and the Archaea)**. Red regions indicate distinct phyla and outlying genomes and groups.

These phylogenies necessarily condense a great deal of information into a relatively simple set of distance scores. Approaches such as Neighbor-Net [60,61] build networks that can represent alternative, incompatible (in the sense of a tree) phylogenetic signals from a distance matrix. Figure 7 (Proteobacteria) and Figure 8 (all other bacterial phyla + Archaea) show the network constructed with the same distance data that were used to build the tree in Figure 6: the full data set could not be analyzed in this fashion due to memory limitations in SplitsTree. Interpretation of this network is subject to the following properties and constraints. First, the network is shown as a cladogram (all edges are of equal length) to emphasize discordance in the data set, and alternative affinities shown in the network typically do not carry the same weight. Second, all splits in this network must be *circularly compatible *[60], which limits the representation to a two-dimensional, planar graph, with the consequence that many alternative affinities may still not be displayed. Finally, as described in [62] and elsewhere, splits graphs do not clearly show "long-distance" affinities between taxa. Nonetheless, relaxing the strict tree requirement can reposition groups and highlight cases where strong disagreement exists.

Although most phyla and classes show similar degrees of cohesion in Figure 7 as they do in Figure 6, certain lineages are positioned in ways that may be suggestive of strongly conflicting signals. For example, the hyperthermophilic bacterial groups Dictyoglomi, Aquificae and Thermotogae are concentrated in one region of the graph, in close proximity to thermophilic members of class Clostridia such as *Caldicellulosiruptor *and *Thermoanaerobacter*, suggesting potential lateral connections between these groups. The Aquificae-delta-proteobacterial association in Figure 6 may reflect a specific linkage between the sulphate-reducing *Desulfovibrio *and the sulphur-oxidising *Sulfurihydrogenibium*. The Spirochaetes are supported by splits that separate them from the other genomes, although *Leptospira *appears to be the most weakly connected member of this group. Based on its reported "vertical" history and lifestyle, *Thermoplasma *might be expected to bridge the Euryarchaeota (its putative phylum based on 16S and concatenated informational protein phylogeny: [7]) and the Crenarchaeota from which it appears to have acquired many genes [63-65]. However, this genus shows no stronger association with the Crenarchaeota than does any other group of Euryarchaeota. Within the Proteobacteria, several outlying groups from the genome trees are also outliers in the network, including the gamma-proteobacterial genera *Francisella *and *Acidithiobacillus *and *S. denitrificans *which remains a close partner with *Arcobacter*. As is the case with *Leptospira*, genomes that flank larger groups in the graph may have particularly strong affinities for other major lineages; examples here in addition to those listed above include *Blochmannia*, the Alpha-proteobacterial genus *Zymomonas*, and the Halobacteria. Other affinities may not be apparent either because they are not reflected in the pairwise genomic distances, or due to the constraints of the neighbor-net approach. To carry out a thorough examination of the affinities of different genomes, a protein-by-protein analysis is necessary.

### Phylogenomic analysis of 1173 microbial genomes

Model-based phylogenetic analysis of orthologous genes is a computationally demanding task. Whereas the analyses above made use of a simple reciprocal best match criterion for inferring orthologous sequences between pairs of genomes, phylogenetic tree construction requires the definition of orthologous sets which can range in size from 1 to 1173 in this data set. It is not sufficient to greedily assemble sets by adding all sequences that share a significant BLAST match at some threshold: Harlow et al. [66] demonstrated that this straightforward approach led to the inclusion of > 87% of proteins in a single, massive "blob", with nonhomologous gene or protein pairs connected via fusion proteins and false-positive BLAST matches. Markov clustering as implemented in e.g., TribeMCL [67] is a popular graph clustering approach that treats the set of matches as a graph by modelling proteins as nodes and each BLAST match between a pair of proteins as an edge connecting those two nodes. The clustering strategy then models the "flow" through different parts of the graph using random walks, and cuts the graph in areas of low connectivity. While benchmarking has suggested that Markov clustering is effective in recovering orthologous relationships, and its time complexity is O(n^2^), making it relatively efficient, is it is non-phylogenetic in nature and in practice does not scale well to graphs with millions of vertices.

An alternative to the graph clustering approach is to assemble sequences into putative homologous sets which can contain > 10,000 sequences in some cases, and use a more-explicitly phylogenetic strategy to subdivide these sets into groups of putative orthologs. BranchClust [68] is an example of such an approach, which allows the user to retain a certain proportion of duplicates from a subset of genomes in a cluster. The approach applied here is similar to BranchClust in that it bases orthology decisions on a phylogenetic tree, but is much stricter in the constitution of orthologous sets. The program UCLUST [22] builds protein clusters at a given level of amino acid identity by iterating through a list of proteins, adding a protein to an existing cluster if it is at least *k*% identical to the "seed" protein for that cluster; if no such identity relationship is found, then the protein is used as the seed for a new cluster. The performance of this algorithm depends on the structure of sequences in the data set: if many similar proteins are present, then many comparisons will be saved, but if all proteins in the set are sufficiently dissimilar then an all-versus-all comparison will still be necessary. A critical advantage of methods such as UCLUST and CD-HIT is the potential to avoid making all-versus-all comparisons; adding more homologs that satisfy the identity criterion to an existing data set will result in a linear, rather than quadratic, increase in the number of comparisons that need to be made.

Since even UCLUST could not process all 3.7 million proteins in a single run, a hierarchical approach based on genome taxonomy was implemented (see Methods); this strategy produced 424,219 clusters with sizes between 1 (orphan proteins) and 21,182. The entire clustering procedure took approximately 30 hours to complete: by contrast, all-versus-all BLAST comparisons for the genome phylogeny analysis above required approximately 70,000 CPU hours. An iterative approach to multiple sequence alignment construction and trimming was performed, based in large part on the use of hidden Markov models to construct accurate alignments with detailed confidence scores. FastTree [24] was used to infer trees from these multiple sequence alignments. Orthologous sequence sets were recovered from the resulting trees by first collapsing groups of in-paralogs, and then by identifying subtrees (clans) in which no genome was represented twice. The procedure of multiple sequence alignment, phylogenetic inference and tree trimming took approximately 90 hours to complete.

**Figure 9:**
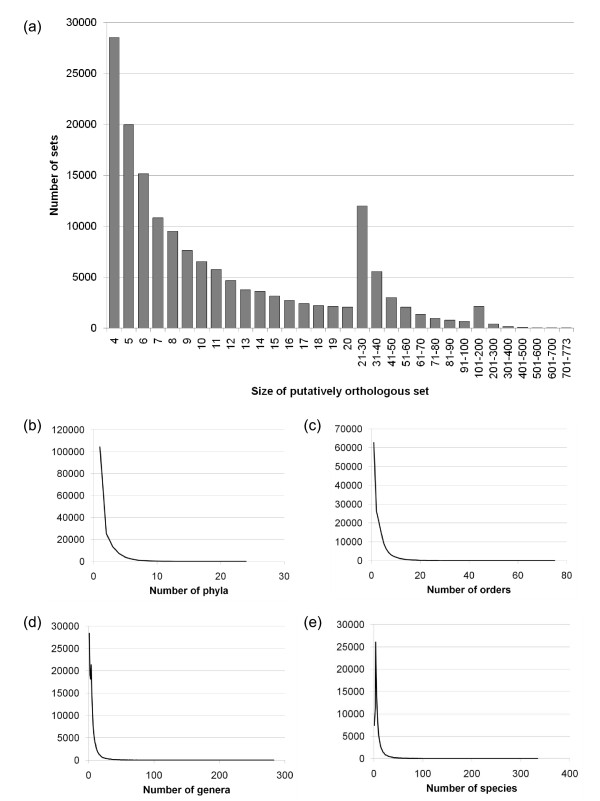
**Frequency distribution and taxonomic coverage of putatively orthologous protein sets of different sizes**. Panel (a) shows the number of orthologous sets based on the number of proteins they contain. Only the retained sets of size ≥ 4, which can generate unrooted phylogenetic trees that are informative, are shown. Panels (b)-(e) show the number of taxonomic groups covered by sets at the taxonomic ranks of phylum, order, genus and species.

A total of 159,905 putatively orthologous sets were recovered using the above procedure (Figure 9a), covering a total of 2,616,080 proteins (68.0% of the original protein set). Of these, 98,175 (61.4%) contain ten or fewer leaves, while only ten trees cover even half of the genomes in the set. The lack of "universal" or "nearly universal" orthologs in this set may reflect failure of the clustering approach to unite distantly related orthologous sets across all bacterial and archaeal phyla, and may also be a consequence of the aggressive approach taken to separate subsets of paralogous sequences. In spite of this subdivision of clusters, Figures 9b, c, d and 9e show that many clusters covered multiple taxonomic groups at every rank. While 104,323 sets covered only a single phylum, over 55,000 sets still contained proteins from 2-24 phyla. A majority of clusters contained sequences from > 1 taxonomic order. The remainder of this work is focused on the recovery of sequences with close affinities to particular taxonomic groups of interest, rather than the construction of a complete summary tree or network; as a consequence the analyses reported below will likely be less sensitive to a failure of the approach to assemble distantly related sequence sets. Focusing on relationships without comparison to a reference "vertical" tree removes the need to express affinities as being either concordant or discordant with respect to some arbitrary reference. Genes with patchy distributions might nonetheless produce trees that do not disagree with a reference phylogeny, even if many intervening taxa are missing and the distribution might better be explained by LGT.

**Figure 10:**
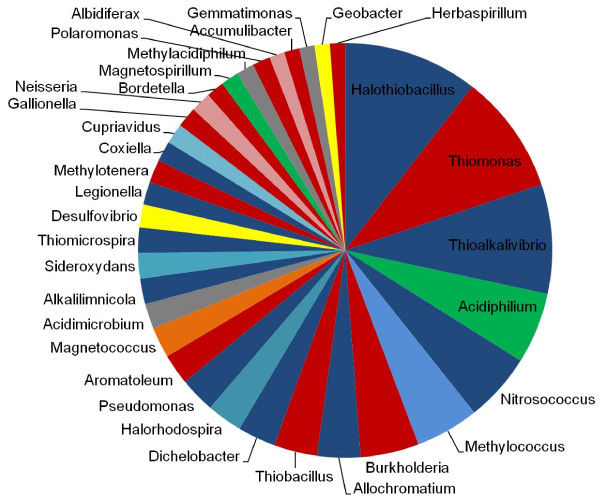
**Taxonomic groups most frequently found in association with proteins from genus *Acidithiobacillus***. The 34 most-frequently observed sister taxa (see Methods for details), covering a total of 504 trees, are shown in decreasing order of co-occurrence. An additional 795 trees in which *Acidithiobacillus *has multiple partners are not summarized, nor are 333 trees covering 179 other partner genera. Blue = Gamma-proteobacteria, red = Beta-proteobacteria, green = Alpha-proteobacteria, yellow = Delta-proteobacteria, orange = unclassified Proteobacteria (*Magnetococcus*), gray = other phyla. Alternating light and dark shades of the same color are used when two or more members of the same group are adjacent to one another in the chart.

A straightforward approach to assess the phylogenetic affinities of a particular group is to identify all trees that contain members of that group (possibly with the additional condition that all members of the group be resolved cohesively in the tree, i.e. a *clan *sensu [69]), and then identify the most likely sister group in each of those trees (see Methods for details). This analysis was performed for the gamma-proteobacterial genus *Acidithiobacillus*, a group separated from the other genomes in its class by the Beta-proteobacteria in Figures 5, 6 and 7. Historically, *Acidithiobacillus *was split from a highly heterogeneous genus, *Thiobacillus*, which was postulated before gene sequencing was widespread but ultimately shown to contain members of several proteobacterial classes when subjected to SSU analysis [70]. The species represented here, *Acidithiobacillus ferrooxidans*, is an obligate autotroph that that can oxidise Fe^2+^, elemental sulphur or thiosulphate and related compounds, and grows at pH < 2.0 [71,72]. A total of 837 trees contained a grouping of *Acidithiobacillus *with a single genus as sister, while an additional 795 trees had multiple genera as the sister group to *Acidithiobacillus*. Figure 10 shows the affinities for the 504 trees covering the 34 most-frequently observed sister taxa; another 179 genera were observed among the remaining 333 trees, but are not shown in this figure. The most common partner of *Acidithiobacillus *is *Halothiobacillus*, another gamma-proteobacterial sulphur-oxidising organism that was removed from genus *Thiobacillus *by [70], while the next most common partner is a sulphur-oxidising Beta-proteobacterium, *Thiomonas*, which was removed from *Thiobacillus *by [73]. The majority of other affinities are to members of these two classes, but genera from other classes such as the acidophilic alpha-proteobacterium *Acidiphilum *are observed, as well as acidophiles from phyla Actinobacteria and Verrucomicrobia. Examination of the functional annotations of proteins from *Acidithiobacillus ferrooxidans *ATCC 23270 indicated that some of the proteins apparently shared with the other genera named above revealed proteins associated with sulphur, but also a wide range of other metabolic (e.g., transaldolase) and informational (e.g., aminoacyl-tRNA synthetase) proteins, as well as transporters and other membrane proteins. Such affinities can be summarized using functional categories of genes, but no class of function appears to be recalcitrant to transfer. Although many partners appear to share ecological properties in common with *Acidithiobacillus ferrooxidans*, the adaptive role of LGT (if indeed it is adaptive) manifests itself in ways much more subtle than simple transfer of obvious pathways, e.g. those directly involved in sulphur metabolism.

**Figure 11:**
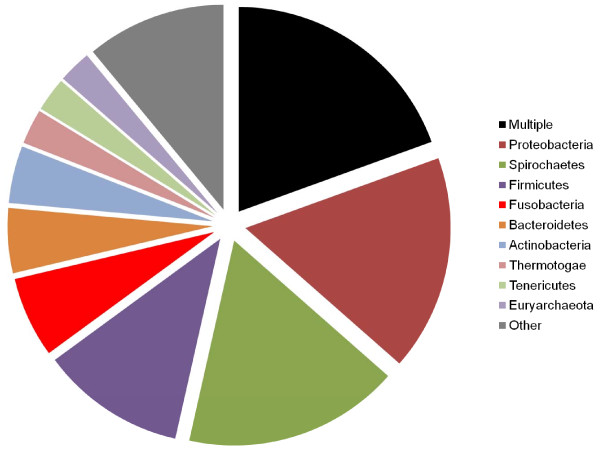
**Phylogenetic affinities of proteins from genus *Brachyspira***. All trees which contained ≥ 1 *Brachyspira *genome in a cohesive grouping (i.e., a clan) are summarized.

As noted above, the position of genus *Brachyspira *in genome phylogenies appears to depend on the inclusion or exclusion of the phylum Fusobacteria: when this phylum is excluded (Figure 6), *Brachyspira *branches with most other members of the phylum Spirochaetes. Representatives of both groups are found together in some habitats such as in adhering to the intestinal mucosa of patients with irritable bowel syndrome [74], suggesting an opportunity for habitat-directed LGT. Individual genes that are similar between *Brachyspira *and *Fusobacterium *have indeed been noted in the literature, including a beta-lactamase [75] and a symporter in close proximity to a mobile element in *Brachyspira *[76]. Figure 11 shows the partners of *Brachyspira*, summarized at the phylum level. The most commonly observed single partner of this genus is in fact the phylum Proteobacteria, which is not suggested by any of the tree or network analyses above. Spirochaetes are second in the list of affinities, followed by Firmicutes and Fusobacteria. Why then does the *Brachyspira*-Fusobacteria association appear in the genome tree of Figure 5? It may be that the similarities between proteins of these two groups (as well as those of Firmicutes) are particularly strong due to recent transfer, with large normalized scores that exert a strong influence on the pairwise genome distances. A breakdown of affinities at the genus rather than phylum level identifies very different key partners: the three most-frequent partners to *Brachyspira *are all Spirochaetes, while the top ten also include one Firmicute (genus *Clostridia*), two Fusobacteria, a genus from phylum *Fibrobacteres*, another Spirochaete genus, one from Tenericutes and finally a lone proteobacterial genus. Although in aggregate, the affinity of *Brachyspira *with Proteobacteria is quite strong, this affinity is spread out over a total of 70 genera.

**Figure 12:**
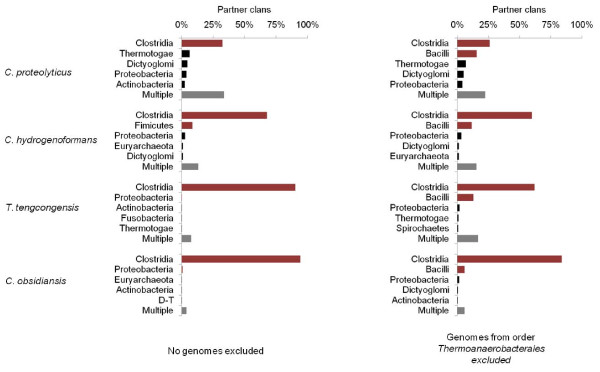
**Most-frequently observed phylogenetic partners of four thermophilic members of class Clostridia**. The proportion of gene trees in which each genome has the indicated phylum or class as its sister is shown. Firmicute classes are highlighted in red, while sister groups comprising multiple phyla are shown in gray.

The position of *C. proteolyticus *in the genome phylogeny was particularly interesting, as this organism was the only member of the relatively well-sampled Firmicutes phylum to branch with members of other phyla. This observation is consistent with the phylogeny reported by [77] which placed two species of *Coprothermobacter *as sisters to *Fervidobacterium *and *Thermotoga*, and the analysis of [58] that placed this organism apart from the other Firmicutes. Other thermophilic Clostridia have been sequenced, including representatives of genera *Thermoanaerobacter, Carboxydothermus*, and *Caldicellulosiruptor*. Several alternative approaches were used to characterize the affinities of *C. proteolyticus*. First, an affinity analysis was carried out at the level of phylum (with Firmicutes subdivided into its two main classes, Bacilli and Clostridia), to determine which of the four clostridial thermophiles had significant affinities to other major groups. *C. proteolyticus *and to a lesser extent *Carboxydothermus hydrogenoformans *(Figure 12) show phylogenetic affinities with Proteobacteria and other groups in addition to Clostridia and Bacilli. By contrast, *Thermoanaerobacter tencongensis *and *Caldicellulosiruptor obsidianus *match the Clostridia almost exclusively. This simple analysis, however, does not consider the extent of sampling effort within the order Thermoanaerobacterales, to which all of these four organisms belong. Indeed, *T. tengcongensis *and *C. obsidianus *both have other congeners present in the data set, dramatically increasing the likelihood that their best matches will fall within order Clostridia due to self-matches at the genus or family level. A second analysis was carried out by removing all non-self members of Thermoanaerobacterales from the trees, and re-running the comparison. Although *T. tengcongensis *and *C. obsidianus *match a greater number of non-clostridial organisms in the second analysis, *C. proteolyticus *and *C. hydrogeniformans *still show much stronger affinities to non-Clostridia and non-Firmicute groups. The strongest non-Firmicute affinities of *C. proteolyticus *in the revised analysis are to phyla Thermotogae and Dictyoglomi, which along with phylum Synergistetes are its closest partners in the genome tree in Figure 5.

**Figure 13:**
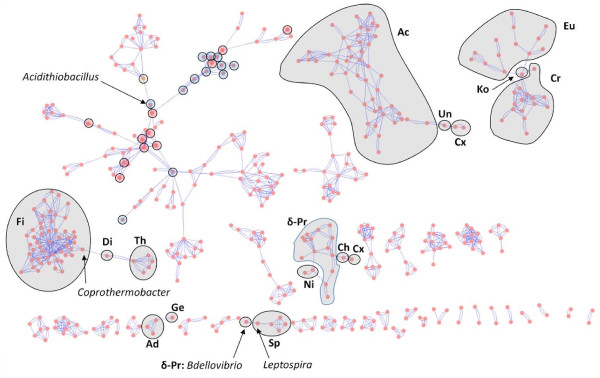
**Intergenomic affinity graph for 455 genera built from 159,905 input trees**. Each pink circle represents a genus, and blue edges connect genera with affinities that satisfy the minimum incidence criteria (see Methods). Circled genera in the largest component correspond to proteobacterial genera identified in Figure 10, with blue, red and green indicating members of the Gamma, Beta and Alpha subdivisions respectively. Clusters that are heterogeneous at the phylum level are indicated with abbreviated names of their constituent phyla: Ac = Actinobacteria, Un = Unclassified Bacteria, Cx = Chloroflexi, Eu = Euryarchaeota, Ko = Korarchaeota, Cr = Crenarchaeota, Ni = Nitrospirae, Ch = Chlorobi, δ-Pr = Delta-proteobacteria, Fi = Firmicutes, Di = Dictyoglomi, Th = Thermotogae, Ad = Acidobacteria, Ge = Gemmatimonadetes, Sp = Spirochaetes.

Holloway and Beiko [78] formalized the structure of an *intergenomic affinity graph *(IAG) which represents each genome as a node in a graph, with nodes connected by edges when some evidence for strong affinity (e.g., BLAST similarity, shared gene content or phylogenetic proximity) is observed. IAGs were previously constructed (although not named as such) by [79-82]. Here the IAG structure is applied to sets of genomes, aggregated at different taxonomic levels, to assess whether named taxonomic groups are indeed cohesive when phylogenetic evidence is considered. Figure 13 shows an IAG based on genus-level affinities from the set of trees generated above. This cluster is similar to the graph generated by [81] using a combination of compositional similarity and gene tree proximity, although the largest component of the graph in [81] contains a much larger number of genomes from Firmicutes and other phyla in addition to the Proteobacteria. A total of 37 distinct clusters were recovered, with some genera not included because their affinities with other genera did not meet minimum threshold requirements (see Methods). Some clusters are homogeneous at the phylum level: phyla Aquificae, Cyanobacteria, Deferribacteres, Deinococcus/Thermus, Fusobacteria, Planctomycetes, Synergistetes, Tenericutes, and Verrucomicrobia each localized to a single cluster which contained members of no other phylum. Large phyla tended to be split across several clusters, with Proteobacteria spanning eleven, Actinobacteria spanning four, and Euryarchaeota four: notably, the Firmicutes were all in a single cluster. Six clusters contained representatives from more than one phylum: the single cluster containing the largest number of Actinobacteria has one genus (*Rubrobacter*) that connects to the unclassified bacterial genus *Thermobaculum*, which in turn is connected to a Chloroflexus (*Sphaerobacter*) and a Chlorobi (*Thermomicrobium*). Notably, all four of these organisms are thermophilic. A single cluster contains all genera from phyla Crenarchaeota and Korarchaeota, along with several methanogenic and other genera of Euryarchaeota. A group of sulphur-metabolizing Delta-proteobacteria is connected to phylum Nitrospirae, a single Chlorobi and the Chloroflexus genus *Dehalococcoides*. All Acidobacteria (three genera) and the lone Gemmatimonadetes genus are connected in a single cluster. The Spirochaetes cluster together, but the unusual genus *Leptospira *is also connected to a single delta-proteobacterial genus, *Bdellovibrio*. Finally, the position of *Coprothermobacter *in this IAG is consistent with previous analyses shown above, since it is connected to the three Firmicute genera *Bacillus, Clostridium*, and *Thermoanaerobacter*, but is also linked to Dictyloglomi, which are in turn linked to all genera in phylum Thermotogae.

The largest cluster in the graph contains the majority of gamma-, beta-and alpha-proteobacterial genera. *Acidithiobacillus *and many of its partners from the analysis shown in Figure 10 are present in this graph; in particular, sulphur-metabolizing organisms and acidophilic genera are connected to one another, with acidophilic genera responsible for the inclusion of Alpha-proteobacteria in this cluster. The diverse genera *Pseudomonas *(9 partners), *Shewanella *(10 partners) and *Burkholderia *(11 partners) are critical hubs in this graph, linking groups that would otherwise be divided into separate clusters. Many of the bridges induced by these and other organisms are taxonomically inconsistent but suggestive of LGT driven by common ecological roles, or arising opportunistically due to presence in the same habitat. Groups that are taxonomically *and *ecologically cohesive tend to share many connections; for example the enteric bacteria which are also found in the largest cluster, connected to the environmental organisms above (*Burkholderia*, acidophiles, etc.) through insect endosymbiont genomes.

**Figure 14:**
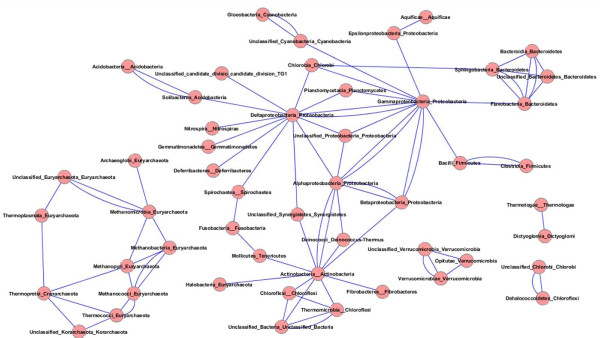
**Intergenomic affinity graph for 53 classes**. Node and edge representations are as in Figure 13; class name and parent phylum are shown for each node in the graph.

An IAG was constructed at the class level to assess higher-order affinities. The resulting graph (Figure 14) contains only five connected components, three of which are relatively small (Dictyoglomi with Thermotogae; three verrucomicrobial classes connected with one another; and a pairing of Chlorobi with class Dehalococcoidetes from phylum Chloroflexi, consistent with the genus-level IAG). The second-largest component contains all Archaeal orders with the exception of Halobacteria, which associate instead with Actinobacteria in the largest component. Notable hubs in the largest component include Actinobacteria, which is connected to eight other phyla; Gamma-proteobacteria, which is connected to three other phyla in addition to all other classes of Proteobacteria; and the Delta-proteobacteria which are connected to a remarkable ten other phyla and three other classes of Proteobacteria. This wide range of affinities may help to explain the unusual placement of this group in the genome phylogeny of Figure 6, and is consistent with the alternative affinities seen in the genus-level IAG. Although different groups within this class are likely responsible for different observed connections, the number of associations seen indicates an important role for this class in LGT to various distantly related groups. Interestingly, the Epsilon-proteobacteria connect only to two other classes: the Gamma-proteobacteria and Aquificae. There is uncertainty about whether Aquificae are "early-branching" thermophiles with affinities to other such groups including Thermotogae, or whether they are close relatives to Epsilon-proteobacteria that have been extensively remodelled through LGT and other evolutionary processes [16,83-85]. The affinities in this IAG certainly suggest no strong association with other exclusively or largely thermophilic phyla. The connection between Spirochaetes and Fusobacteria may be driven by the affinities of *Brachyspira *shown above.

**Figure 15:**
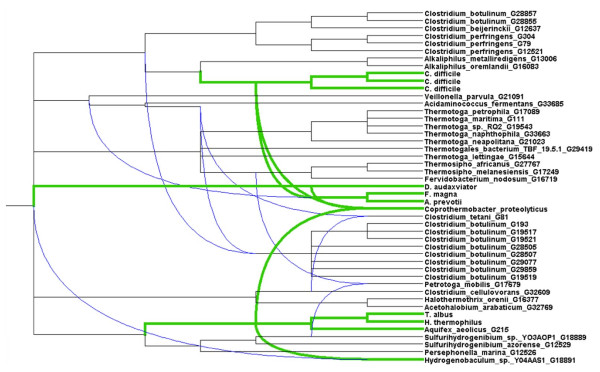
**Galled network centered on *C. proteolyticus***. Straight lines represent tree edges, while reticulation edges are shown with curved lines. Lineages with close affinities to *C. proteolyticus *are highlighted in green.

Given the diverse set of conflicting relationships exhibited by many different microbial groups at all levels of taxonomic classification, it is evident that a genome tree will necessarily fail to adequately describe the complex web of relatedness seen in prokaryotes. Furthermore, the susceptibility of tree approaches to artifacts arising from conflicting signals [55] can produce groups in the tree (clades, clans, etc.) that are potentially supported by none of the data. Network methods are an obvious remedy to the limitations of tree approaches, and several new techniques and algorithms [25,61,86-88] have been proposed. Two particularly promising algorithms are galled networks and cluster networks [89], which can explicitly show reticulate connections between otherwise distant taxa without introducing an exhaustive set of splits between them. Furthermore, these approaches require no reference tree for comparative purposes, unlike reconciliation or "HGT/LGT" networks [90-92]. A disadvantage of these approaches is that all input trees must be rooted: given a set of trees covering variable sets of taxa, with potentially rampant LGT and variable rates of evolution disrupting any consistent pattern that might be expected, it is difficult to justify any rooting strategy that might be proposed. Still, if these algorithms can be further refined, they will provide an avenue by which complex intergenomic relationships can be visualized in a phylogenetic context (as opposed to the non-phylogenetic IAGs shown above). Figure 15 shows a cluster network built with Dendroscope [93], which shows connections among taxa with previously reported affinities to *C. proteolyticus*. Trees including this species as well as representatives from Dictyoglomi, Thermotogae, Aquificae and optionally other Clostridia were selected, with the additional requirement that Archaea be represented for rooting purposes (although interdomain transfers are certainly possible). Only 13 trees satisfied these taxonomic requirements: the relevant RefSeq identifiers and annotated functions for proteins from *C. proteolyticus *are shown in Table 1. The functions span a wide range of activities (core metabolism, DNA repair, replication, etc.) and are quite different from the transcriptional and translational proteins that are typically used to produce reference trees. At the chosen threshold (see Methods), the cluster network identifies three distinct partners for *C. proteolyticus*. Within class Clostridia, *C. difficile *and a group comprising *Desulforudis, Finegoldia*, and *Anaerococcus *are connected to *C. proteolyticus*, while affinities with phylum Aquificae are also seen, although notably to only a subset of members of this phylum. Inspection of the 13 input trees, however, suggests that some important relationships are missed by the cluster network: notably, four of the 13 input trees have *C. proteolyticus *in close association with either Dictyoglomi or Thermotogae or both of these phyla, an association that is not seen in the galled network.

**Table 1:** RefSeq identifiers and annotated functions of *C. proteolyticus *proteins included in the cluster network of Figure 15.

RefSeq GI	Annotated function
206895185	MoaAnifBpqqE family protein

206895224	Dihydropteroate synthase (dihydropteroatepyrophosphorylase)

206895314	Phosphoribosylformylglycinamidine synthase II

206895389	Fructose-1,6-bisphosphate aldolase, class II

206895496	Anaerobic ribonucleoside-triphosphate reductase activating protein

206895498	Dimethyladenosine transferase

206895580	Formate--tetrahydrofolate ligase

206895681	DNA mismatch repair protein

206896085	Replication factor C subunit

206896220	Phosphate transport system regulatory protein PhoU

206896247	S-adenosylmethionine:tRNA ribosyltransferase-isomerase

206896383	Hypothetical protein

206896487	Hydrogenase expressionformation protein HypE

## Conclusions

### Telling the whole story

There is no shortage of "genome trees" in the literature. Inferred trees have been explicitly claimed as a "Tree of Life" [8,14], as a null hypothesis of vertical descent to be taken seriously [16,18] or rejected as ludicrous given the amount of disagreement in the data [33], and completely bypassed based on a lack of strong statistical support for any particular structure [94]. Also notable is the disagreement about the extent of LGT, which will vary depending on the specific data set analyzed, the analytical technique used (e.g., identification of phylogenetically discordant sequences, gene content or tree comparisons), and the method used to count LGT events. Strong statistical support has been used to argue that recovered relationships should be treated as canonical, but simulation studies have shown that robust statistical support can be assigned to phylogenetic relationships that are incorrect [55]. It is evident from the analyses performed above and elsewhere that strict hierarchical relationships must not be taken at face value, but examined carefully to determine which components of the genomic data, *if any*, are consistent with the reported relationships.

It is evident too that traditional concatenation approaches are not adequate to the task of dealing with highly reticulated relationships. Informational proteins appear in lists of transferred genes, with implied transfer distances (e.g. between proteobacterial classes, or bacterial phyla) that are unlikely to be explainable due to artifacts of phylogenetic inference. If the probability of transfer of any particular gene is nonzero, then adding genomes will only diminish the candidate set of "transfer-free" genes; as soon as a set is contaminated with LGT examples, incorrect topological relationships are likely to emerge [95]. Since the purpose of concatenation is to reinforce weak phylogenetic signals that cannot be robustly recovered from any single gene, the concatenation approach would seem to be particularly vulnerable to the effects of conflicting signals in the data. Techniques such as Concaterpillar [96] can address discordant signals up to a point, but still need some degree of cohesion among the input alignments as a basis for building consistent sets.

Using all of the available data (or, more precisely, all data that can be examined using a particular approach) can reveal a multitude of alternative affinities. Even the genome trees in Figures 5 and 6 reveal conflict in two ways: in comparison with each other, and through comparison with SSU-based microbial taxonomy. The repositioning of certain groups such as the Delta-proteobacteria, Aquificae, *Brachyspira*, and Mollicutes hints at a conflicting set of relationships (based only on pairwise distances between genomes!) that cannot be represented in a single tree, and the odd positioning of *Acidithiobacillus, C. proteolyticus *and other lineages relative to their taxonomic partners suggests a potential disconnect between SSU rDNA and other important components of the genome. The phylogenetic network in Figures 7 and 8 introduces two-dimensional relationships to accommodate some amount of incongruence, but the conclusions that can be drawn from inspection of such a heavily reticulated network are limited to a general confirmation of discordance in the data, and a possible tendency for particularly heterogeneous lineages to fall at the fringes of their taxonomic groups. The highlighted genera in Figure 7, and loosely connected assemblages in Figure 8 (e.g., Spirochaetes, *Thermoplasma*, and the two main Firmicute divisions) again hint at multiple attractions, but cannot confirm specific identities. Clearer examples of networks (i.e., with fewer reticulations) are presented in [61] and elsewhere; these rely on less discordance in the data or more-aggressive filtering criteria. The galled network in Figure 15 has the potential to show a much clearer picture of the complex relationships among lineages, but imposes constraints on the input trees that are not reasonable when rates of LGT are high.

Affinity graphs set aside questions of phylogeny in favour of capturing strong relationships among a set of input taxa. The connections in these graphs can be generated in a number of ways, such as sequence identity of encoded proteins [80], optimization of best BLAST matches [78], surrogate patterns of LGT [81] or sister relationships in phylogenetic trees ([81]; this work). By expressing a genome as a set of affinities, connections can be recovered without recourse to tree rootedness and the "verticality" or "laterality" of relationships. This approach not only identified groups that appear to be most strongly influenced by LGT such as the thiosulphate-metabolizing bacteria, *C. proteolyticus *and *Leptospira*, but guarantees that connections in the graph are supported by some component of the input data.

The case of the thiosulphate-reducing acidophiles, exemplified by *Acidithiobacillus*, gives a particularly compelling historical example of the tortuous relationship between taxonomy, phylogeny, and ecology. The original genus *Thiobacillus *was split into several genera across three proteobacterial classes in recognition of both the ecological and metabolic diversity of these organisms, and the story told by SSU rDNA analysis [70]. However, LGT seems to be reinforcing the cohesion of these groups, with many trees suggesting transfer between *Acidithiobacillus *and other acidophiles. While some of the > 200 genera with which *Acidithiobacillus *showed strong affinities may represent artifacts, the picture of the evolution of these groups is undoubtedly complex.

A similar pattern is emerging for thermophiles such as *C. proteolyticus*. Thermophiles have been previously identified as substantial exchangers of genes to the point where some thermophiles such as *Aquifex aeolicus *and *Thermotoga maritima *appear to be hybrids comprising significant numbers of genes from lineages as diverse as the Epsilon-proteobacteria, thermophilic Clostridia, and the Archaea [16,85,97]. *C. proteolyticus *is a thermophile and a member of the Clostridia, a group noted elsewhere for its frequent exchange of genes with other groups [98,99]. The resulting genetic composition of this organism is sufficiently complex that it appears connected in many summaries to other phyla.

Klenk and Göker [27] suggested that sequencing microbial genomes for taxonomic breadth rather than specific industrial, ecological or medical reasons will break long branches and reinforce genomic relationships in a Tree of Life. However, the results for acidophiles, thermophiles and other groups suggests otherwise: rather than revealing a clear and "true" pattern of affinities among lineages, new genomes may simply increase the patchwork of affinities without yielding further insights into the evolutionary origins of major lineages (e.g., the patterns of cellular division and inheritance that might provide a framework for hypotheses of vertical descent). Aggregation techniques will combine these data and generate trees that are "largely congruent with the SSU tree", at the cost of hiding a great deal of conflicting information. While it is true that extremophilic lineages may represent a worst-case scenario for the recovery of vertical relationships, when one considers the effect of setting aside various types of extremophiles, and the large uncertainty at deep phylogenetic levels (which is likely to be sensitive to even a small number of LGT events), one must ask what relationships remain that can be confidently asserted above the genus or family level.

### ...with very large data sets

Taken together, the pan-genome (Figure 3) and affinity analysis (Figure 4) indicate that even within a single named species or genus, there is a great deal of gene content variation and affinity variation for constituents of the "core" genome. This variability makes it difficult to defend the exclusion of sequenced genomes from a full analysis, at least without an initial pre-screening phase. Holloway and Beiko [78] proposed the use of an efficient first-pass technique to remove genomes with no affinities to the groups of interest; if the genus-level affinity screen of *Acidithiobacillus *is to be taken as an example, then one might discard all genera not present in the list of > 200 with high affinities, or even filter more aggressively as was done in Figure 10. Since many alternative affinities shown in Figure 4 involve disagreement at the species level or finer, a screening pass might be used to remove congeners whose affinities outside of their own genus are similar to other congeners that are retained. Aggregation of species or genus pan-genomes would be another option, removing most members of homologous sets that have consistent affinities with other lineages (e.g., ribosomal proteins).

Even with simplifications outlined above, a set comprising > 10,000 sequenced genomes covering > 1000 genera (a likely outcome of sequencing efforts such as GEBA, and the rapidly diminishing costs of sequencing technology more generally) will still present a formidable analytical challenge. One can imagine a series of phylogenomic approaches that progressively sacrifice precision and optimality in favour of reduced computation time. A wholly impractible strategy would be to use the guaranteed optimal (for a given scoring scheme) Smith-Waterman [100] algorithm to do an exhaustive pairwise comparison of all protein sequences, greedily aggregate into large "blobs", and then use exact alignment methods [101] and exhaustive likelihood-based tree search approaches to generate massive trees within which orthologous sets (or mostly orthologous sets) can be identified. The alternative approach used in this work required less than one week of computation on a single CPU, and could conceivably scale well to much larger data sets using a combination of filtering as above, parallel processing and by exploiting repeated structure in the protein sequence data.

While the approach used here supports very rapid analysis of large sequence data sets, it currently sacrifices too much precision to support a full phylogenomic analysis, as demonstrated by the failure to recover any ubiquitous or nearly ubiquitous orthologous sequence sets. Several refinements to the approach, particularly at the level of orthology inference, need to be explored. The rate of false negatives (i.e., pairs of proteins that had a BLAST match at or better than a given threshold, but failed to end up in the same cluster, was quite high even at relatively high levels of stringency: for example, of the 440,173,605 pairs of proteins that matched with a BLAST e-value of 1 × 10^-50 ^or less, only 250,974,989 (57.0%) ended up in the same cluster. This number is strongly influenced by the way in which matches are counted: for example, splitting a cluster containing 20 proteins into two subclusters of size ten would yield  true positives, and 100 false negatives, for an overall sensitivity of 47.4%. Nonetheless, full orthologous set recovery will require the development of approaches that still avoid the quadratic scaling of all-versus-all comparisons, while retaining more information about clustered sequences. One approach with considerable potential is the hierarchical amalgamation of proteins into a profile or hidden Markov model representation, which would aggregate many proteins into a single statistical summary for comparative purposes instead of discarding all but one member of the cluster.

An obvious alternative to *ab initio *inference of orthologous relationships is to start with an existing reference database of putative orthologs or homologs such as OMA [102], MBGD [103], EggNOG [104] or OrthoMCL [105] as a scaffold, and overlay new sequences to the existing reference sets. This can be accomplished using either complete homology search against the reference database, or by building statistical models for each of the reference database families and assigning new sequences to the set that corresponds to the best-matching model. Such approaches will however depend on the scaling of the original algorithm used to build the reference database: in cases where the clustering approach requires all-versus-all comparisons as an initial step, constructing sets on 10,000 genomes may not be feasible.

A persistent challenge in the analysis of these data sets lies in the reporting of relationships, particularly in mapping these relationships into a tree or network. A widely used technique for static visualization of trees with many leaves is to collapse cohesive named groups (as was done in Figures 5 and 6), or to color leaves according to some taxonomic, genomic or ecological criterion. This strategy, and interactive techniques for expanding and collapsing clans or clades, and focusing on particular parts of the tree [93,106] are effective when the relationships shown are hierarchical and strictly bifurcating. However, network representations generate additional challenges for layout and interpretation: indeed the full Neighbor-net of 1173 genomes could not be inferred and visualized in SplitsTree due to processor and memory limitations. It is evident too that the sacrifice of clarity made by Neighbor-net to allow some conflicting relationships to be shown, does not produce an effective visualization for entire microbial genomes. Cluster networks, reconciliation networks and IAGs are better in this regard as they make explicit connections between taxa with strong affinities, but the resulting structure can saturate with alternative connections: even the tree in Figure 15, which is based on only 13 trees covering a subset of all taxa, contains 12 reticulations in addition to the underlying multifurcating tree. Interference between these edges limits the interpretability of the static image. The key to extracting the desired information from network representations will be in effective use of data screening, thresholding and filtering techniques prior to building the network, and the use of interactive focus-plus-context [107] approaches to emphasize key relationships.

## Methods

### Genomes and system used in this study

A total of 1180 completely sequenced prokaryotic genomes, containing a total of 3,849,772 predicted protein-coding genes were acquired from NCBI on September 29, 2010. A complete list of genomes can be obtained by filtering the page http://www.ncbi.nlm.nih.gov/genomes/lproks.cgi to include only those genomes released up to that date. To restrict this set to those genomes collected in complete years, a subset of 1052 genomes released up to December 31, 2009 was also assembled. Assignments at the taxonomic levels of superkingdom (= domain), phylum, class, order, family, genus, and named species were also collected from NCBI: in cases where a label was undefined at a given level, the value of the next-highest level was propagated downward with the addition of the prefix "Unclassified".

All analyses for which times are reported were carried out on a 360-CPU-core Linux cluster consisting of Intel Xeon X5460 processors with 3.16 GHz clock speed and average 4 GB of RAM per core. Times reported are approximate as there may have been small performance losses due to network latency on the cluster and competition for disk and network access with other running processes.

### Assessment of protein inventories and affinities

Comparative protein inventories of sequenced genomes were first assessed using a string comparison to extract matches at the 100% identity level. Distinct sequences remaining after this analysis were subjected to an all-versus-all BLASTP comparison using the BLAST+ package, version 2.2.23, with default parameters used apart from an expectation value threshold of 1 × 10^-3^, and the use of soft masking of low-complexity regions. From this set, different analyses were performed using more-stringent expectation value thresholds.

Novel proteins were identified in a temporal fashion as follows: starting with genomes sequenced in the year 1995, all proteins were assessed to determine whether they had a BLAST match better than a specified threshold of 1 × 10^-10 ^to a protein already in the set. Proteins lacking homologous matches at this threshold were considered to be novel proteins and used to increment the total count of homologous sets seen thus far. Proteins matching no other protein in the database were labelled as orphans.

Pan-genome analysis required the definition of homologous sets of sequences with an indication of which sets were represented in which genomes. For each genus with at least ten sequenced representatives, homologous sets were built by considering the proteins encoded by each genome in turn. Each protein encoded by the initial genome was compared against each other protein from the same genome, and those pairs with BLAST e-value ≤ 1 × 10^-5 ^and local alignments covering at least 70% of the length of the matching sequence were merged into the same homologous set. Each subsequent genome was compared against the existing homologous sets, with matching proteins added to the appropriate set, and proteins with no match used as the seeds for new sets. After the final genome was added in this way, a complete set of phylogenetic profiles, indicating which homologous sets cover which genomes, was obtained. A single pan-genome analysis for a given genus was performed by choosing *k *reference genomes at random, and then assessing the effect of adding a randomly chosen genome *k *+ 1. The percentage gain in homologous sets from the addition of genome *k *+ 1 was expressed as the ratio of proteins in novel homologous sets in the newly added genome, to the total number of proteins present in the first *k *genomes. This procedure was repeated ten times for each combination of genus and value of *k*, and values were summarized by computing the mean and standard deviation of the ten replicates.

The affinities of congeneric and conspecific genomes were assessed by first partitioning their encoded protein sets into shared (core) and variable components. A protein from a genome was marked as shared if it had at least one BLAST match to a protein in the other genome satisfying the minimum sequence similarity criteria (e-value ≤ 1 × 10^-5^, local alignments covering at least 70%). Closest affinities were then assessed by finding the best BLAST match (smallest e-value) between each protein from the genome and the rest of the database, including its partner at the genus or species level, but excluding all other members of the same genus or species. For each genome in a pair, its affinity profile was computed as the proportion of its core proteins that had a best match to the partner genome and other non-self lineages. The affinity difference is equal to the Euclidean distance between these profiles. All pairs of congeneric organisms were from different named species.

### Calculation of genome distances and construction of genome trees and networks

Genome-level analyses were based on the computation of distances between each pair of genomes in our set. These distances were computed as described in [108], by first identifying reciprocal best-matching (RBM) proteins and then computing a normalized bit score for each such RBM pair *a, b*. The normalization was computed as follows:

where *S_a, b_*is the bit score of the BLAST match using sequence *a *as query and *b *as subject. The calculation therefore divides the lesser score between the two proteins by the greater of the two protein self-scores. Seven small genomes failed to return any RBMs when compared against certain distantly related genomes; these small genomes were removed from the set, leaving a total of 1173 genomes. For the reduced set, all genera having two or more genomes were identified. The largest genome from each such genus was added to the set, as was the largest genome from a different named species within the same genus, if available. For genera with only a single representative named species, the second-largest genome from the same species was instead added to the set.

Genome trees were inferred using the FastME application, version 1.1 [109] with default parameter settings. Networks were constructed using the Neighbor-net algorithm as implemented in Version 4.11.3 of SplitsTree [61]. The "use weights" option was disabled to allow the construction of reticulated cladograms; otherwise all default parameters were used.

### Phylogenomic analysis

Detailed phylogenomic analysis was performed using a pipeline of hierarchical inference of putative homologs, multiple sequence alignment with quality checking, and phylogenetic inference with trimming to isolate putative sets of orthologs. While UCLUST [22] is computationally efficient and uses relatively little memory, *ab initio *clustering of > 3 million sequences is not possible with the freely available 32-bit implementation. UCLUST was therefore first applied to sets of genomes aggregated at the taxonomic level of order. All protein sequences from genomes associated with a given order were collected into a single multiple-FASTA file, sorted in decreasing order of length and then clustered with UCLUST version 3.0.617 using an identity threshold of 60% for clustering. This procedure was then repeated at the phylum level, using only those proteins that were retained as cluster seeds in the first round of clustering. The phylum-level cluster seeds were then compared against one another using all-versus-all BLASTP with a maximum e-value threshold of 1 × 10^-10^. Phylum-level seeds that matched at this level or better were used to build master sets, and at this stage all of the clustered proteins that were removed at the order or phylum level were restored to their respective clusters. The resulting sets contain putatively homologous proteins. This procedure yielded a total of 424,219 separate clusters containing a total of 3,849,448 proteins and 1,216,174,052 amino acid residues.

Multiple sequence alignment was carried out using an iterative process, based on MUSCLE [110] and HMMER 3.0 [111], that yielded quality scores useful in trimming uncertain positions and entire sequences from alignments. Three steps were performed, with repetition as necessary, to generate high-quality alignments: (i) initial alignments were constructed using the recommended "fast" settings of MUSCLE (-maxiters 1 -diags -sv -distance1 kbit20_3); (ii) these alignments were used to build hidden Markov models in HMMER using the "hmmbuild" program with default parameters; (iii) sequences were realigned to the HMM using the "hmmalign" program. This last step generates quality scores ranging from 1 to 10 for each residue in each position of the alignment, and these were used to trim uncertain regions from the alignment. First, the average quality score for all residues of each sequence was calculated. Any sequence with an average quality score less than 9.0 was removed from the set. Removal of one or more sequences from the alignment in this fashion triggered a reanalysis of the remaining sequences starting with a new MUSCLE alignment. When no further sequences were removed in this fashion, average quality scores were then computed for each column of the alignment, and any column with an average quality score < 8.0 was removed. Any data set with fewer than 4 remaining sequences or 50 alignment columns was not subjected to phylogenetic analysis. At the end of this step, 107,696 clusters (25.4% of all original clusters), 3,052,033 proteins (79.3%), and 1,033,204,827 amino acid residues (85.0%) remained. The low retention of initial clusters is likely due in large part to the elimination of clusters of size 1, 2 and 3, along with protein sequences too short to satisfy the minimum length criterion.

Phylogenetic inference was performed using FastTree version 2.1.0 [24]. FastTree uses the Jones-Taylor-Thornton amino acid substitution model by default; we retained this and all other default parameters, except for those recommended by the authors to improve the effectiveness of the tree search at a slight cost of increased running time (flags: -spr 4 -mlacc 2 -slownni). Many of the resulting trees contained > 1 sequence for at least one genome in our set, and needed to be pared down to build sets of putative orthologs by subdividing the tree into *representative *subtrees [66] in which no genome is represented more than once. The first step in this procedure was to identify clans [69] that contained sequences exclusively from a single genome; these sets were treated as in-paralogs, and all but one of the sequences in this clan were removed. Any trees that still had > 1 sequence from one or more genomes after this step were subdivided into representative subtrees by identifying the largest representative subtree, i.e. the largest subtree with a maximum of one protein from any given genome. These subtrees were iteratively pruned from the larger tree in decreasing order of size until the remaining tree was itself representative, or until the largest candidate subtree for pruning had fewer than four sequences. These pruning procedures were implemented using the Phylo library in BioPython [112].

Taxonomic affinities for particular groups *G_0_* of interest were recovered by identifying the subsets of inferred trees containing a clan *C_0 _*which contained all sequences from *G_0 _*and no sequences from any other taxonomic group. Unless the tree contained only one sequence not from *G_0_*, the internal edge separating *G_0 _*from all genomes not in *G_0_* is adjacent to two other edges which define clans *C_1_* and *C_2 _*which cover genome sets *G_1 _*and *G_2_*. These two clans are candidate sister groups for *C_0_*: placing a root anywhere within *C_1 _*would make *C_2 _*the sister group to *C_0_*, and vice versa. Whichever of *C_1_* and *C_2_* contained the largest number of leaves was used to root the tree, thus choosing the smaller clan as sister to *C_0_*. If the selected sister clan contained only genomes from a single group at a particular given taxonomic level, the count of partners between *G_0 _*and the group represented by the sister clan was incremented by one. If the sister clan was heterogeneous, then the count of partners containing multiple groups was instead incremented by one. The phylogenetic inference and pruning step yielded 159,905 clusters containing a total of 2,616,080 proteins (68.0% relative to the initial clusters) and 873,468,715 amino acid residues (71.8%).

An intergenomic affinity graph (IAG: [78]) summarizes strong affinities between individual genomes or higher-level groupings by representing each entity (genome or group) as a vertex in a graph. Edges between vertices indicate evidence for evolutionary affinities. IAGs in this analysis were built by aggregating genomes at the genus and class levels, and searching the set of inferred trees for examples where two classes were cohesive (i.e., clans) and adjacent in a tree; edges were created between vertices if certain minimum threshold criteria were satisfied. The first criterion was established by identifying the strongest affinity (largest number of observed sisters to any nonself group) for each group *G_i_*, and requiring all other candidate partner groups for *G_i_*to have at least *k*% as many edges as this strongest connection. For example, if genus *Pseudomonas *had the genus *Cellvibrio *as its closest partner in 100 trees, then *k *= 20% would require other candidate genera to be partnered with *Pseudomonas *in at least 20 other trees to generate a link between the two genera in the IAG. In practice this led to genera represented by very small genomes becoming hubs in the IAG since their maximum affinity count might be less than 5, and any other group with at least one connection would then be attached to this genus. An additional minimum count requirement *c *was added to further filter the set of results: no affinity observed fewer than *c *times would be included in the IAG, even if the count otherwise satisfied the *k*% requirement. At the genus level, *k *was set to 20% and *c *set to 5, while at the class level *k *was set to 10% and *c *to 5.

Galled networks were constructed using Dendroscope version 2.7.4 [93]. Since the full set of trees could not be combined into a single graph due to algorithmic and visualization constraints, subsets of trees were selected to address particularly the relationship between *C. proteolyticus*, its presumed closest relatives based on 16S rDNA analysis (class Clostridia), and candidate partner taxa identified in previous analysis, specifically phyla Aquificae, Dictyoglomi and Thermotogae. Trees were subselected based on the requirement that members of all these groups were present. Additionally, since these network algorithms require rooted trees as input, selected trees were further required to contain at least one representative from domain Archaea to serve as an outgroup to the bacterial sequences for rooting purposes. Although such a scheme will be compromised by interdomain LGT, the recovered affinities of *C. proteolyticus *may not be impacted if the LGT events are not "close" to the target genome, i.e. the implied partners in an interdomain transfer are not ancestors of or sisters to *C. proteolyticus*. Since input trees covered overlapping but non-identical sets of genomes, the Z-closure procedure [113] was used to impute missing taxa prior to network construction. A threshold of 0.65 was used for network construction: above this level, many relationships were collapsed into multifurcating nodes and no reticulations were present, while a much larger set of reticulations appeared when thresholds of 0.50 were used.

## Reviewers' comments

### Reviewer's report 1

Joel Velasco, Cornell University, USA

Overall, I think that this is an exciting and interesting paper well worth publication. There is lots of careful, foundational discussion on how large-scale genome phylogenies can be done as well as a presentation of a variety of options for the future and this is very valuable. The analysis itself is also interesting and important. I have nothing in particular to say about potentially surprising results for the locations or affinities of particular groups though I do want to point out the especially important results about not just the volume of reticulation, but the high level of reticulation between specific non-sister groups.

A few specific comments on the paper with quotes from the author followed by my own comments:

Page 8:"Some newly annotated proteins are 100% identical to an existing protein, in which case only one or the other needs to be included in a sequence alignment or phylogenetic analysis".

That depends on your methods and goals. For example, if you are trying to build a phylogeny of genomes, you definitely want to include these identical genes as they are very strong evidence for which genomes are sisters.

#### Author response

Indeed, all I meant was that you don't need to run MUSCLE or RAxML on identical sequences. I have reworded to the following:

*Some newly annotated proteins are 100% identical to an existing protein, in which case only one or the other needs to be included in the sequence alignment and phylogenetic inference steps, while the others can be restored in the appropriate location in the final tree or network*.

Page 8:"Using a simple BLAST threshold criterion, a total of 418,214 homologous sets of proteins was identified; over half of these (255,417) were orphan proteins."

What is a "homologous set of proteins"? Is one set a set of proteins all of which are homologous to each other? In that case, none of these could be orphans since to be an orphan is just to NOT be homologous to any other protein in a particular set. I guess you mean that the proteins were divided into equivalence classes based on homology and the orphans were thus in sets of size one. If so, this makes sense, but the language is a bit confusing. Also, you should be careful here, "a simple BLAST threshold criterion" probably means something like > x% sequence identity but this is not transitive and so what happens if A+B meet the crierion and B+C do, but A+C don't?

#### Author response

*If we include a trivial self-homology criterion in the definition of homologous sets, then I think that orphans can be said to be in homologous sets of size 1. In any case, I have hopefully removed the ambiguity by adding "...in homologous sets of size 1" to the end of the offending sentence. See further discussion on this matter below*.

*The transitivity question is a vexing problem, but in this case I used a temporal approach to identifying homologs, as described in the second paragraph of "Assessment of protein inventories and affinities" in the Methods section, so transitivity is not an issue. It's a subtle distinction, but I'm not actually building sets here: as long as you match any protein that has already been seen, you're not the seed for a new homologous set. The method doesn't care about the specific protein that was matched*.

Page 9:Discussion of Figure 3 (and so relevant for the caption of Figure 3 on page 55).

I am a bit confused by exactly how you got the numbers in Figure 3. You say on page 9, "homologous sets of proteins" and later "gene family count" and the section ends with "novel genes". In the caption on page 55:"Percent increase in pan-genome size (number of previously unseen homologs vs. total number of homologs)..." But what is a homolog? Homology is a two-place relational term. So an unseen homolog makes no sense (to be a homolog of anything, you have already seen what it is homologous to). It could mean a gene which is homologous to another gene which we previously thought was an orphan. But this is not an increase in the size of the pan-genome, but a redundancy. I assume you mean to say the number of previously unseen genes divided by total genes previously known. This meaning is closest to the pan-genome idea of your citation [35]. But "novel genes" should be distinguished from novel gene families and from novel proteins ([36] explicitly uses proteins instead of genes in their measure of novelty but then discusses the "proteome"). A novel protein can come from a previously known gene and a novel gene can come from a previously known gene family. Also, it should be clear whether "novel" means just novel within the group, or the discovery of a unique gene which is not previously known anywhere. These come apart because of LGT. The former is typically the relevent one for pan-genome discussions, but in the context of discussing large-scale phylogenies (including multiple genera) "uniqueness" probably means unique relative to all genes in the analysis.

Terms like "homolog" and "homologous gene set" are used throughout the text in numerous places and so if they don't make sense here, they should be changed throughout.

#### Author response

*First, "gene family" was a mistake: I have corrected the single instance of "gene family" to "homologous set", which should be clarified by the added definition of homologous sets*.

*Second, since homology is a transitive criterion (setting aside the thorny question of fusion proteins and partial homology), I think it is reasonable to generalize the idea of a homologous pair of genes/proteins to a homologous set in which each gene/protein is homologous to all the other genes/proteins in the set. This forms the intuitive basis for phylogenetic profiling and other techniques. I have changed a few instances that referred to the presence or absence of a particular "homologous protein" (with the hanging question of "homologous to what?"), to refer instead to the presence/absence of a protein from a particular homologous set*.

I now provide this definition on p.9:

*A homologous set is defined theoretically as a group of one or more proteins in which each protein is homologous with every other protein in the set; such sets are assumed to be maximal, in the sense that all homologous pairs of proteins are assigned to the same homologous set. In practice, empirical, putative homologous sets inferred from sequence data rarely satisfy both of the above criteria, since homology is not always detectable from sequence similarity, and fusion proteins (among others) produce "partial homology" relationships that make perfect sets impossible to define*.

*Orphan proteins are defined immediately afterward as being members of homologous sets of size 1*.

*The Figure *3* caption has also been changed:*

Percent increase in pan-genome size (number of proteins assigned to previously unobserved homologous sets vs. total number of proteins)

Page 9:"Genera with large standard deviations such as Clostridium and Mycobacterium have such internal structures".

I do believe that these groups have internal structure, but not merely because of variance. As you point out, biased sampling in a group alone will cause variance. If the tenth genome sampled just happens to gain no new genes, you will get variance (if the others do have new genes). The internal structure of the group could be anything you like-say, a genus with 3 species. On the other hand, what to my mind is a more structured group-say a genus with 10 different species and one sample from each species with each equally spaced in genetic distance from the rest-you will tend to get about the same benefit out of any of the 10^th ^genomes and so there will be very little variance.

#### Author response

I think this is what I was trying to say. I have added further illustration to the Clostridium/Mycobacterium example to clarify this:

*Genera with large standard deviations such as Clostridium and Mycobacterium have imbalanced internal structures as the sampling effort is dominated by a few pathogenic species such as C. botulinum and M. tuberculosis, and genomes in both groups have a wide range of gene counts: different amounts of novelty will result if the tenth added genome is M. leprae (1284 genes) or M. smegmatis (3490 genes)*.

Page 10:"There is consequently no reason to suspect that the rate of accumulation of novel genes will decrease in the near future".

I don't understand this sentence. There is diminishing marginal utility in the sense that each new genome in a group brings less novelty than the previous genome. Similarly, genomes from new groups aren't as valuable as new groups were previously due to just plain old vertical history but besides that, LGT dictates that the more samples you have, the more incoming and outgoing LGT genes you will have. So you could say that there is no reason to suspect that we will cease to discover new genes, but you can't say that the rate of accumulation won't decrease. Of course it will decrease unless the diminishing returns is continually compensated for by increased rates in sequencing. Now this may well happen, but the context seems to indicate that this exponential growth in sequencing isn't playing a role in the claim.

#### Author response

That's exactly what I meant, and I have reworded to clarify the point:

*Given the amount of novel genetic information in new genomes and the increasing rate at which genomes are being sequenced, there is consequently no reason to suspect that the rate of accumulation of novel genes will decrease in the near future*.

Page 10:"Broad generalizations based on SSU data are only valid if SSU rDNA faithfully tracks organismal and genomic patterns of inheritance and relatedness."

This depends on how broad the generalization is and exactly what counts as faithful. For example, if rRNA doesn't perfectly track organismal history (and so is not faithful), it can still be a reliable basis for some broad generalizations (e.g. LGT is more common between groups X and Y than between X and Z, archaea and bacteria diverged very deeply in the past, etc.)

#### Author response

*Changed "if" to "to the extent that"*.

Page 11:"localized reticulations that reflect phylogenetic uncertainty."

I am not sure how a reticulation can represent uncertainty. Unresolved nodes can certainly do this. Reticulations in a network can indicate that genes that have discordant histories and so in some sense uncertainty as to the organismal phylogeny, but they aren't really representing uncertainty, but actual evidence of real reticulation.

#### Author response

*Actually, reticulations can indeed represent uncertainty or ambiguity in a relationship: see for instance Ho SYW, Jermiin LS (2004) Tracing the Decay of the Historical Signal in Biological Sequence Data. Syst Biol 53:623-637. They are particularly useful in cases where you can't settle on A+B or A+C in a tree, but can definitely rule out A+D*.

Page 12:"The genomes from species *Actinobacillus pleuropneumoniae *and *Actinobacillus succinogenes *have stronger affinities to members of genera *Haemophilus *and *Mannheimia*, respectively, than they do to each other." (and other related sentences from pages 11+12).

I am confused by Figure 4 and the broader discussion of the whole issue on pages 11 and 12. Take Figure 4a. In the caption on page 56, you say that the data comes from a pair of genomes of the same species. But then what does it mean for a shared protein to be a best blast match to a different partner? Does it mean that at least one member of these pairs is a better match to something else? Or that each of them is individually a better match to something else? For example, A might be the best blast match to the conspecific B, but B's best match might be C. I would assume that this would be the most common case. For example, if conspecific A+B form a clade for a given gene and then there is a transfer between B+C, B+C will now show the affinity, but A will not now be closer to something else. Given this, how is the affinity difference calculated?

#### Author response

*There are two distinct concepts here, neither of which was explained in sufficient detail in the earlier version of the Methods. For a given pair of conspecific or congeneric genomes, one can ask whether their proteins tend to match one another most frequently. Even if they do not, one genome might still be able to serve as a valid proxy for the other if their overall pattern of taxonomic matches is similar. The affinity differences shown in *Figure 4* are based on the dissimilarity (Euclidean distance) between the match profiles of one genome vs. the other. This value is large when proteins from genome 1 tend to match lineages A, B, C and D, while proteins from genome 2 match to lineages E, F, G and H*.

I have added two sentences in the Methods to clarify this:

*For each genome in a pair, its affinity profile was computed as the proportion of its core proteins that had a best match to the partner genome and other non-self lineages. The affinity difference is equal to the Euclidean distance between these profiles*.

On page 11, you say, "The two members of *Chlorobium phaeobacteroides *show different affinities to other members of their own family: 53.2% of encoded proteins in *C. phaeobacteroides *strain BS1 match best to genus *Prosthecochloris*, whereas proteins in strain DSM 266 tend to best match the congener *C. limnicola *(39.0%) and the closely related genus *Pelodictyon *(32.4%)." First, 53.2% of what? Total encoded proteins or just those that are shared with its conspecific partner? Second, the two numbers of the DSM 266 strain add to over 70% which looks to be higher than the data point on Figure 4a. This can be reconciled depending on what that data point represents, but this really needs to be spelled out.

#### Author response

*First, 53.2% of all non-ORFan proteins. Reworded*.

*Second, the data point in *Figure 4a*is dependent on the overall profile similarity of the two genomes, rather than the percentage of proteins from each genome that do not have a protein from the other genome as their best match*.

On page 12 you seem to be assuming there is some kind of symmetry here where if A is close to B, its congeneric C is close to some other D. Or maybe all of the examples just happen to have this feature, but it is a bizarre and unlikely one. If A and B were sharing a lot of genes, A's congeneric C would naturally be close to both A and B. But in all the mentioned examples, there seems to be a new fourth group involved. Is there a biological explation for this surprising find?

#### Author response

*No such assumption is made-indeed the reciprocal affinities for the two Chlorobium cases are quite different, with strain BS1 having a stronger affinity for DSM than vice versa. The point of the affinity differences is to test a weaker assumption, namely, even if two congeners are not each others' best matches due to LGT, can one nonetheless still tell a similar "story" as the other due to similar affinity patterns with other lineages? In the cases described here, the answer is clearly no*.

Page 30:"However, the results for acidophiles (and thermophiles, see e.g. [16]) may suggest otherwise: rather than revealing the true affinities of lineages, new genomes may simply increase the patchwork of affinities and confuse the accurate recovery of evolutionary origins."

The broader point is correct, that lots of extra genomes will increase the number of affinities and in theory, this might not help. But more data probably will help. However you deal with discordance in the first place, say concatenation, can in some cases be a bad idea, but the expectation is that more data works better. If it actually leads you away from the truth, this is the case where the new data is actually misleading evidence (possible). But again, the solution to misleading evidence is to have more evidence. If the new data leads to less confidence in the answer we thought was good before, there is a very good chance it was misleading evidence before. And in any case, the overall evidence being inconclusive is probably what we should think if we are in such a case (we happen to have had it right before, but we can't know that is the case). Smaller data sets in some cases can lead us to be more confident of a particular answer due to less discordance, but they are more likely to lead us to be confident in the wrong answer. This being said, there are serious computational and practical reasons that including more data is not always better. But those aside, more data is better. -- At least, it is clear that this is what happens if the model of LGT is that there is a vertical signal which we are trying to detect and then a bunch of other conflicting signals due to horizontal transfers. If we are trying to do more than recover the vertical signal and don't want to hide conflicting data, etc. then more conflicting data which is at a weaker strength is actually worse.

#### Author response

This is an excellent point, and the original sentence was poorly phrased. I was trying say that more data are not likely to reinforce the notion that phylogenetic patterns of microbial evolution are knowable and/or easily recovered, if only we had representatives of Lineages X, Y and Z. Hopefully the following wording clarifies the point:

*...rather than revealing a clear and "true" pattern of affinities among lineages, new genomes may simply increase the patchwork of affinities without yielding further insights into the evolutionary origins of major lineages (e.g., the patterns of cellular division and inheritance that might provide a framework for hypotheses of vertical descent)*.

### Reviewer's report 2

William Martin, Heinrich-Heine-Universität, Germany

This reviewer commented on the manuscript, but did not provide a response for publication.

#### Author response

This review, although not for publication, was helpful and led me to make the following changes to the manuscript:

- *Addition of several new references*

- *Correction of grammatical/punctuation mistakes*

- *Important changes to the Abstract regarding the thrust of the entire article*

- *Explicit reporting of % of retained/discarded sequences*

- *The addition of panels b-e in *Figure 9, *and a short discussion of these*

I also provided a different interpretation of the clustering pipeline, which is not suitable for the main manuscript but complements the description in the Methods section:

*(1) Proteins that are at least 60% identical (which is quite similar) to a "seed" protein are temporarily removed from consideration. We retain a set of seeds at the order level*.

*(2) These seeds are compared with one another at the phylum level, at the same 60% identity threshold. Again, seed sequences are identified, and sequences matching these seeds are removed*.

*(3) The phylum-level seeds are compared to one another using BLAST, at an e-value threshold of 10^-10^. Sets of seeds that satisfy this criterion are grouped into a single cluster, and then all of the matching sequences that were removed in steps 1 and 2 are put back*.

*As long as the proxies are able to represent the clusters they cover, then no information is lost in the progression up from lower ranks to higher ones. With the preliminary UCLUST-based approach I used here, however, the high level of false negatives indicates that quite a bit of information was lost by reducing sequence sets to proxies at the order and phylum level. This is one of the reasons why I emphasize the need for more-sensitive methods than the simple "proxy protein" approach presented here. Even so, the new *Figure 9c* shows that many clusters span quite a few orders/phyla, even after the filtering steps below are carried out*.

*The motivation here is not to highlight the taxonomy, but rather to exploit the fact that hierarchical aggregation will work best if it can toss out many similar proteins earlier in the process. Since taxonomic groups do show some degree of gene content cohesion, the choice to aggregate in taxonomic terms is intended to exploit this property*.

### Reviewer's report 3

Eugene V. Koonin, National Center for Biotechnology Information, NIH, USA

Review of "Telling the Whole Story in a 10,000-Genome World" by Robert G. Beiko

This is a highly impressive study by virtue of the sheer number of trees analyzed (> 150,000). Much of the article is devoted to overcoming the genuinely formidable technical difficulties that hamper phylogenomic analysis on this scale. These problems emerge at every step, from the identification of orthologs to tree or network visualization.

Under these circumstances, I found the presentation of the Methods lacking. In my view, all the procedures need to be described with a considerably greater precision, in order to assess the true utility of the approaches described in the article. From what I did glean from the Methods, I am rather concerned with the robustness of the identification of orthologous sets. Although the two-step approach employed here-clustering first, then FastTree-seems to be quite reasonable, the clustering procedure is very restrictive, so there are likely to be many false negatives, and it is unclear how many there are. So it is uncertain to what extent the results are impacted. The author recognizes the problem but does not offer a remedy. I wonder whether it would make sense to use existing clusters of (putative) orthologs like EggNOGs as seeds, then assign new sequences to these seeds, then use FastTree to refine, and only then identify new (rather small) clusters among the remaining sequence de novo.

#### Author response

*Concerning the presentation of the Methods, I hope that my responses to the previous referees clarifying the calculation of genomic affinity differences (i.e., Figure *4*), the taxonomic coverage of the final orthologous sets (see *Figure 9b-e*) and the number of proteins and residues retained at each step of the pipeline (see Methods) gives some further clarity. In addition to this, I have given a characterization of the level of false negatives in the second half of the Discussion, which further illustrates the aggressive subdivision of clusters*.

Basing clusters on existing orthologous sets is a viable strategy, but relies on these algorithms themselves being scalable and robust. Any algorithm that requires an all-vs-all comparison to begin with, is going to fail when genomic databases get sufficiently large. I have added a paragraph to address this:

*An obvious alternative to ab initio inference of orthologous relationships is to start with an existing reference database of putative orthologs or homologs such as OMA *[102], *MBGD *[103], *EggNOG *[104]*and OrthoMCL *[105]*as a scaffold, and overlay new sequences to the existing reference sets. This can be accomplished using either complete homology search against the reference database, or by building statistical models for each of the reference database families and assigning new sequences to the set that corresponds to the best-matching model. Such approaches will however depend on the scaling of the original algorithm used to build the reference database: in cases where the clustering approach requires all-versus-all comparisons as an initial step, constructing sets on 10,000 genomes may not be feasible*.

The article does not have far reaching biological conclusions beyond the somewhat trite point that evolutionary relationships in such a complex, huge set of genomes are...well, complex. This is understandable and might not be a deficiency in itself as the paper is primarily methodological, a proof of principle in a sense. However, the title of the paper seems to imply that the whole story is indeed told here, which is certainly not the case.

#### Author response

The main aims of this paper are:

- *To critically examine the validity of proxy-based approaches in phylogenomic inference by quantifying the diversity and disagreement that we know about know now, and to project what might happen in the future;*

- *Given the complexities that are present, to assess the extent to which modern computational methods are able to handle a rapidly expanding genomic data set, and to propose an analytical framework that can scale well with increasing data set sizes;*

- *Given the degree of discordance that results (and that has been described elsewhere, although not in much depth for the groups I focus on), assess the strengths and limitations of different visualization approaches*.

*Since the specific pipeline I used needs further investigation and refinement, and since none of the groups of interest was comprehensively explored, there is indeed no basis for claiming "far reaching biological conclusions" from this work. However, the Acidithiobacillus/acidophile story in particular provides an interesting insight into the relationship between trait-based, genome-based and 16S-based taxonomy, echoing earlier results from thermophiles and emerging results from some habitats (particularly the human and other animal-associated microbiomes). Anyone who wants to claim that 16S-based taxonomy is useful in this context will have to address the disconnect with other key genomic and phenotypic properties of these organisms*.

### Reviewer's report 4

W. Ford Doolittle, Dalhousie University, Canada

This impressive paper is a bit like prokaryotic genome evolution itself: amazingly complex and with tantalizing hints of underlying order, but the only certainty is in the details. I'm not competent to critique the methods Prof. Beiko employs here, but do want to add a few comments, mostly about words and concepts and the shifting nature of the connections between systematics and phylogenetics. I see this paper as pivotal to a renegotiation of these connections, and it would be interesting to know if Prof. Beiko does too.

There was systematics before Darwin and there will be systematics after LGT. What Darwin did was claim that systematics, properly understood, is phylogenetics. That natural classifications are naturally hierarchical because phylogenetics is about lineage bifurcation is half of his theory. But if phylogenetics-by which I mean the generation of phyla, not the making of trees-is more complicated than that, then there is no uniquely justifiable way to convert phylogenies to classifications, which become once again a matter for negotiation, not discovery.

"Once again" is especially appropriate in the microbial context because-as Woese has reminded us repeatedly-before 16S rRNA became the "universal molecular chronometer", microbial systematists had given up on any larger evolutionary taxonomy and were learning to be content with identification being the only realizable goal of classification. What LGT means is that identification may now also be the only justifiable goal.

But the community at large has yet to accept that, and Prof. Beiko seems a bit reluctant to come right out and say it himself, in this otherwise admirable essay. Instead he still speaks of LGT as something which "complicates our efforts to assess the degree to which different organisms are related" rather than as something which defines their relationships. Probably many people doing phylogenetics, whether seeking to make trees or nets, still believe that there is something we might call a true phylogenetic position for any species or strain or individuals, and that is a matter for discovery, not negotiation.

But once we accept genomic mosaicism (and Prof. Beiko's figures, especially Figure 10, provide stunning illustrations), we are obliged to ask which genes count most in determining this position and whether a preponderance of genes disagreeing with rRNA is enough to trump its claim. Indeed we could ask whether we might allow phenotypic characters (and especially medically relevant ones) of the sort on which Bergey's Manual of Determinative Bacteriology was based in the pre-rRNA days, back in the door. There are instances in which such characters are more predictive of broader clinical phenotype than is phylogenetic position based on trusted markers (for example Ogura et al. 2009, Proc Natl Acad Sci USA 106:17939-44), and in the metagenomic and human microbiomic communities the notion that taxonomic inventories are less useful than surveys of functional gene repertoires has gained wide acceptance. Once again, we can hope for useful guides to identification but a truly natural hierarchical classification is not achievable, in practice or principle.

#### Author response

*I maintain, as I did in Biology & Philosophy last year, that the tree of cell divisions is a worthwhile construct because it reflects the need for some degree of evolutionary and ecological continuity from one generation to the next. This tree is the proving ground for LGT, because obviously not all genes can be successfully transferred (= acquired, integrated, and fixed via drift or selection) into all lineages at all times. Even if every gene has been transferred at least once in its long history (a proposition I can easily accept), most of its existence will nonetheless consist of boring old vertical inheritance. So the success of an LGT event will depend on the cell's current genetic, genomic, physiological and ecological state, with most states prohibiting most transfers from occurring. Further to these constraints, one needs to consider the differential effects of "buying" a gene via LGT relative to merely "renting" it: in the latter case, a gene is of negligible or only transient benefit and is relatively quickly shunted into the processes of loss that have been documented by, for instance, Weilong Hao and Brian Golding. This is in a way reminiscent of the idea of LGT as global "churn" put forth by the papers of Charles Kurland in the early 2000s. Although I think the Kurland work is entirely too dismissive of the importance of LGT, the core idea is nonetheless worth thinking about: if we can distinguish bought genes from rented ones, then we might refine our ideas about the long-term impact of LGT on prokaryotic groups*.

*I don't expect this approach to suddenly give us a magical Tree of Cells/Life that solves the problems of phylogenetic classification-indeed, given the degree to which LGT events in Acidithiobacillus appear to be Haphazard Orthologous Replacements of Random Stuff, the effect of distinguishing bought vs. rented might not get us too far in that direction. It might, though, remove a lot of edges in an inferred network that are supported by very few genes, if such events correspond to neutral "LGT rain" and the resulting genes are on the way to destruction. Even if we don't get closer to a clear history of cellular lineages, such a refinement might allow us to focus on network features that correspond to LGT highways that are at least partially adaptive*.

*But I think that we can, to some extent, use a structure that is mostly tree-like to relate at least some lineages back to a certain point. I say "to some extent" because some sets of genomes like those of acidophiles or thermophiles may prove to be hopeless cases (although many more potential relationships will nonetheless be rejected than supported by LGT). I say "mostly tree-like" because recombination obviously messes things up dramatically at the tips, and because of recent work by Jeffrey Lawrence that demonstrated the difficulty (impossibility?) of establishing clean bifurcations within enteric bacteria due to protracted periods of speciation. Finally, "back to a certain point" reflects the problems of signal saturation due to excessive genetic change, the effects of bias and other phenomena that are not directly linked to LGT, along with the possibility that LGT has basically homogenized gene content since a particular time in the ancient past. Chasing these answers is worthwhile, but is probably not going to yield a taxonomy that can account for everything unless we are willing to accept a certain amount of imprecision or statistical guesswork*.

*So is trait-or phenotype-based classification the answer? From a utilitarian standpoint, sure, especially when you consider the possibility that microbe X with functions Y today can pick up new traits via LGT and suddenly become able to do Z. Using genetic or microbiological diagnostics that target the trait of interest rather than, say, 16S, will stand a chance of picking up this novel change. But such definitions will be necessarily utilitarian and pluralistic: for the purposes of defining "natural" taxonomic ranks and relationships, I think the tree of cellular divisions is the right choice. The problem, of course, is that the ToC is unlikely to be completely knowable*.

*Apart from changes I have made in response to other referees' comments softening a perceived verticalist stance, I have not brought this discussion into the main manuscript. This is in part because there is some duplication with my 2010 paper in Biology and Philosophy, and in part because I think it deviates too far from the main aims of the paper, which is to quantify and acknowledge the complexities of the data, and to propose viable pathways to support their analysis. The questions you raise are fundamental, and I think a key component of moving this work forward is that we need to acknowledge that what we need may exist, but the nature of the molecular data may not allow us to know it in its entirety*.

#### Doolittle response in a second review

Either we don't really disagree or are agreeing to. It's actually hard to think of any evolutionary biological argument that does not to take the form that the TOL/TOC debate has taken. First we all endorse model A, then model B is cast in opposition to it, then it is accepted that both have merit, but efforts are made to quantify their relative applicabilities-with A-ists and B-ists each trying to bias the results in their favor. Then (though we are not quite there yet) we come to realize that there is no agreeable-upon measure of relative strength. He and I would agree that it is possible (but not proveable) that no gene in any genome is the product of an unbroken lineage of vertical descent events dating back 3.8 billion years, and that it is probable (even proven) that very few are. This to me is total victory for us "enthusiastic lateralists". But he and I would also agree that vertical descent events outnumber LGTs a gazillion to one, and that much can be learned by mapping events (LGTs and other) to trees made from core genes, seemingly a capitulation to the camp of "committed verticalists".

##### Author response

*Part of the problem lies in casting the debate as a metaphorical Agincourt between two opposing camps-the teeming hordes of Charles VI who are verticalists by default or by intention, and the outnumbered and besieged band of Henry V who seek to overturn the existing order. One can even imagine a hand gesture for the lateralists to use after the battle-index and middle fingers on one hand raised and crossed by the index finger from the other hand. But the simple fact of the matter is that four billion years is a long time, and it takes very little in terms of bias and confounding factors to mess it up, whether or not there is an 'it' to begin with. That a convincing Tree of Life cannot be recovered from molecular sequences should come as no surprise, nor should the fact that shallower relationships can indeed be interpreted to some extent as cohesive lineages against which ecological and phylogenetic hypotheses can be tested*.

So what it boils down to mostly is the meaning of words like "relationship" and "natural" (with respect to classification), and whether we think higher taxa are anything but reified conveniences. And maybe there is one substantive quibble, about the Tree of Cells. Any model we accept for the origin of eukaryotes will violate treeness (which I take to preclude anastomoses), as will the primary and any number of secondary plastid endosymbioses. And then we have to admit that quite large chunks of DNA can be transferred between prokaryotic cells of different "species" by conjugation, which involves a sort of cell fusion, albeit temporary. So really even the Tree of Cells has issues.

##### Author response

*I don't want to get into an argument about whether mitochondria have free will, but it seems to me that by the time the prokaryote has cast off its last pretenses of being a free-living organism in its own right, it's pretty obvious to see who's in charge. Which is to say that any sort of lateral event implicating members of different "species" according to some undefined biological species concept, is necessarily going to be asymmetric and require that one of the participants take primary responsibility for outer membranes, cytokinesis, exchange of nutrients and metabolites with the environment, defence, and so on. These types of event are unquestionably of tremendous evolutionary significance, and the contributions of both partners are of obvious importance to genotype, phenotype and role of any successor lineages. But I think the inherent asymmetry in these events allows a particular kind of Tree of Cells to remain as a construct*.

## Competing interests

The author declares that he has no competing interests.

## Authors' contributions

RGB designed and executed the analyses, and wrote the manuscript.
